# A new genus and eight new species of the subtribe Anillina (Carabidae, Trechinae, Bembidiini) from Mexico, with a cladistic analysis and some notes on the evolution of the genus

**DOI:** 10.3897/zookeys.352.6052

**Published:** 2013-11-19

**Authors:** Igor M. Sokolov

**Affiliations:** 1 Department of Entomology, California Academy of Sciences, Golden Gate Park, 55 Music Concourse Drive, San Francisco, CA 94118, USA

**Keywords:** Coleoptera, Adephaga, Anillina, *Zapotecanillus*, new genus, new species, southern Mexico, Isthmus of Tehuantepec, forest litter, identification key, cladistic analysis, syntopic speciation, allopatric speciation, biogeography

## Abstract

One new genus and eight new species of anilline carabids are described from southern Mexico. The new genus, *Zapotecanillus* **gen. n.**, is established for *Z. oaxacanus* (type species) **sp. n.**, *Z. nanus* **sp. n.**, *Z. iviei* **sp. n.**, *Z. ixtlanus* **sp. n.**, *Z. montanus* **sp. n.**, and *Z. kavanaughi* **sp. n.** from the Sierra Madre de Oaxaca, *Z. pecki* **sp. n.** from the Sierra Madre del Sur, and *Z. longinoi* **sp. n.** from the Sierra Madre de Chiapas. A taxonomic key for all described species of *Zapotecanillus* and a cladistic analysis, based on morphological data, are provided. Morphological, behavioral and biogeographical aspects of the speciation in the genus obtained from the resulting cladogram are discussed.

## Introduction

The anilline fauna of Mexico remains extremely inadequately investigated in spite of numerous publications on carabids of the region. Monographs by Jeannel, revising the world fauna of Anillina, contain no information on Mexican representatives (Jeannel 1937, 1963a, b). To date, only two species from two different genera: *Mexanillus sbordonii* Vigna Tagliantiand *Geocharidius zullini* Vigna Taglianti, have been recorded from Mexico (Vigna Taglianti 1973). The genus *Mexanillus* Vigna Taglianti (1973) was established for beetles that were collected in caves and closely resembled troglobitic trechines in several specialized features and peculiar habitus. The genus *Geocharidius* Jeannel had been established 10 years earlier by Jeannel (1963a) for a Guatemalan species, *Geocharidius integripennis*, described by H. W. Bates (Bates 1882) in his grand “Biologia Centrali-Americana”. Because Jeannel’s description of *Geocharidius* was insufficient, Vigna Taglianti (1973) re-described the genus on the basis of the two species, *Geocharidius integripennis* and *Geocharidius zullini*, known to him at that time. At present, *Mexanillus* is a monospecific genus, whereas *Geocharidius* includes 6 species (Lorenz 2005), five of which are limited in their distributions to Guatemala (Erwin 1982).

Preparing a review of the *Geocharidius* species, I determined that anilline specimens from Oaxaca and, in part, from Chiapas, which were identified mostly as *Geocharidius* by different entomologists, actually belong to the undescribed genus. This paper presents the results of a taxonomic study of this genus.

## Materials and Methods

**Material.** This study is based on examination of 150 specimens of a new genus, representing nine species, eight of which are described as new. The material was borrowed from and/or deposited in the following institutions, identified in the text by the following associated codens:

CAS California Academy of Sciences, 55 Music Concourse Drive, San Francisco, California, U.S.A. 94118 (D. H. Kavanaugh, Curator)

CMNC Canadian Museum of Nature, Entomology, P.O. Box 3443, Station D, Ottawa, Ontario, Canada K1P 6P4 (R. S. Anderson, Curator)

CMNH Carnegie Museum of Natural History, Pittsburgh, Pennsylvania, U.S.A. 15213 (R. L. Davidson, Collections Manager)

KUNHM University of Kansas Natural History Museum, 1345 Jayhawk Blvd., Lawrence, Kansas, U.S.A. 66045-7593 (Z. H. Falin, Collections Manager)

MTEC Montana Entomology Collection, Montana State University, Bozeman, Montana, U.S.A. 59717 (M. A. Ivie, Curator)

NMNH Department of Entomology, United States National Museum of Natural History, Smithsonian Institution, Washington, D. C., U.S.A. 20013-7012 (T. L. Erwin, Curator)

Verbatim label data are given for type specimens of all newly described taxa, with label breaks indicated by a slash (“/”). In a case of series of KUNHM specimens with the same geographical labels but differing in various barcode numbers only, these numbers were replaced in the text by periods of ellipsis.

**Measurements.** All specimens were measured electronically using a Leica M420 microscope equipped with a Syncroscopy AutoMontage Photomicroscopy system (SYNCROSCOPY, Synoptics Ltd.). Measurements for various body parts are encoded as follows: LH = length of head, measured along midline from anterior margin of labrum to the virtual line, connecting posterior supraorbital setae; WH = width of head, at level of anterior supraorbital setae; WPm = maximal width across pronotum; WPa = width across anterior angles of pronotum; WPp = width across posterior angles of pronotum; LP = length of pronotum from base to apex along midline; WE = width of elytra, at level of 4th umbilicate setae; LE = length of the elytra, from apex of scutellum to apex of left elytron; SBL = standardized body length, a sum of LH, LP and LE. SBL measurements are given in mm; others are presented as nine ratios: mean widths-WH/WPm and WPm/WE and body parts-WPa/WPp, WPm/WPp, WPm/LP, WE/LE, LE/SBL, WE/SBL and LP/LE. All values are given as mean ± standard deviation.

**Illustrations.** Digital photographs of the dorsal habitus of new species were taken with the AutoMontage system using a Leica M420 microscope. Line drawings of selected body parts were made using a camera lucida on an Olympus BX 50 microscope or grids on a Labomed Lx400 compound microscope. Scanning electron micrographs were made either with coating on a LEO 1450VP SEM or without coating using low vacuum mode on an ESEM FEI Quanta 200.

**Dissections.** Dissections were made using standard technique. Genitalia were dissected from the abdomens of specimens previously softened in boiling water for 20–30 minutes. Contents of the abdomen were cleared using boiling 10% KOH for 2–3 minutes to remove internal tissues, and then washed in hot water before examination. After examination, genitalia were mounted on plastic transparent boards in dimethylhydantoin formaldehyde resin (DMHF) and pinned beneath the specimen. In some species, investigation of body parts was undertaken in the following way. The whole specimen was cleared, using boiling 10% KOH for ~5 minutes, then washed and dissected in the typical way. Disassembled body parts from one specimen were placed on plastic transparent board, properly oriented, mounted in DMHF and pinned together with the specimen labels.

**Type material.** I had no opportunity to investigate the type material of the Mexican species of Anillina described by A. Vigna Taglianti, so, *Mexanillus sbordonii* is known to us only by the original description. The concept of *Geocharidius* used here, is based on the investigation of a long series (>20 specimens) of *Geocharidius integripennis* (Bates) (Terry Erwin’s identification) from the Quiché Department of Guatemala, which is not the type locality of the species (the latter is located within neighboring Totonicapán Dept.); but these specimens exhibit features that closely match diagnostic features of the genus, mentioned in the literature (Jeannel 1963a, Vigna Taglianti 1973). Types of the Guatemalan species of *Geocharidius* described by T. L. Erwin in his revision of Central American Bembidiini (Erwin 1982) were examined.

**Terms.** Terms used in the paper are largely of general use and follow the literature (Ball and Bousquet 2000; Ball and Shpeley 2005, 2009; Erwin 1974; Jeannel 1963a; Shpeley and Ball 2000), except those for ventral surface structures, terms of which follow the Handbook of Zoology (Lawrence et al. 2010).

**Species ranking.** Species recognition is in accordance with our previous approach (Sokolov et al. 2004).

**Arrangement of taxa in the text.** Taxonomic treatments of species in the text follow mostly the geographical basis. The species sequence starts with the type species, and each following species is more distant from the latter geographically, and, presumably, genetically. Within the Sierra Madre de Oaxaca the sequence generally corresponds to the virtual movement along the Tuxtepec – Oaxaca road in SW direction.

**Descriptions.** The scheme of descriptions follows that of Ball and Shpeley (Ball and Shpeley 2005, 2009).

**Maps.** Maps were downloaded from the web-site: http://www.maps-for-free.com/ and adjusted with the help of Photoshop software.

**Cladistic analysis.** Morphological data were used to reconstruct the phylogenetic relationships among species of *Zapotecanillus*. The analysis was based on the assumption that the ancestral lineage of Mesoamerican anillines was represented by a true litter-dwelling, but not endogean, species. Accordingly, as outgroup taxa, two litter species from the anilline genera *Nesamblyops* and *Geocharidius* were chosen for analysis. The geographically proximate *Geocharidius phineus* Erwin from Guatemala represents the globose species of the genus and is confined to the litter of mid-altitudinal forests (Erwin 1982). Geographically distant *Nesamblyops* sp. from New Zealand, because of the presence of rudiment eyes is considered to be close to the ancestral type of normally blind Anillina (Moore 1980). Furthermore, molecular data, although scarce, suggest that this genus forms a branch on the phylogenetic tree of Trechitae basal to the European and American genera of Anillina (Maddison and Ober 2011). A total of 32 binary or multistate characters (29 parsimony informative) were derived from the external morphological features (22), male genitalia (9), and female genitalia (1) (see Tables 1 and 2 in the Appendix). A character matrix was generated using NEXUS Data Editor 0.5.0. for Windows (Page 2001), and the analysis was performed using PAUP* version 4.0 (Swofford 2002), with heuristic tree searches using random addition sequences (100 replicates), holding 10 trees at each step, swapping on all trees, and excluding parsimony-uninformative characters from the data sets. Character states were treated as unordered and unweighted. Bootstrap analyses (Felsenstein 1985) were conducted with resampling at 1,000 replications using the previously mentioned settings. Branch support was also examined using Bremer support indices (Bremer 1994), calculated using the TreeRot.v3 software (Sorenson and Franzosa 2007).

## Taxonomic treatment

### Zapotecanillus gen. n.

http://zoobank.org/CA8A1E66-49BA-4ADC-9587-E8C210CCA380

http://species-id.net/wiki/Zapotecanillus

#### Type species.

*Zapotecanillus oaxacanus* sp. n., by present designation.

#### Etymology.

The name *Zapotecanillus* derives from the Zapotecs, the name of the indigenous people living in the territory of Oaxaca during historic times, and the generic name *Anillus* Jacquelin du Val, the type genus of the subtribe.

#### Recognition.

**Figures 1–4.**
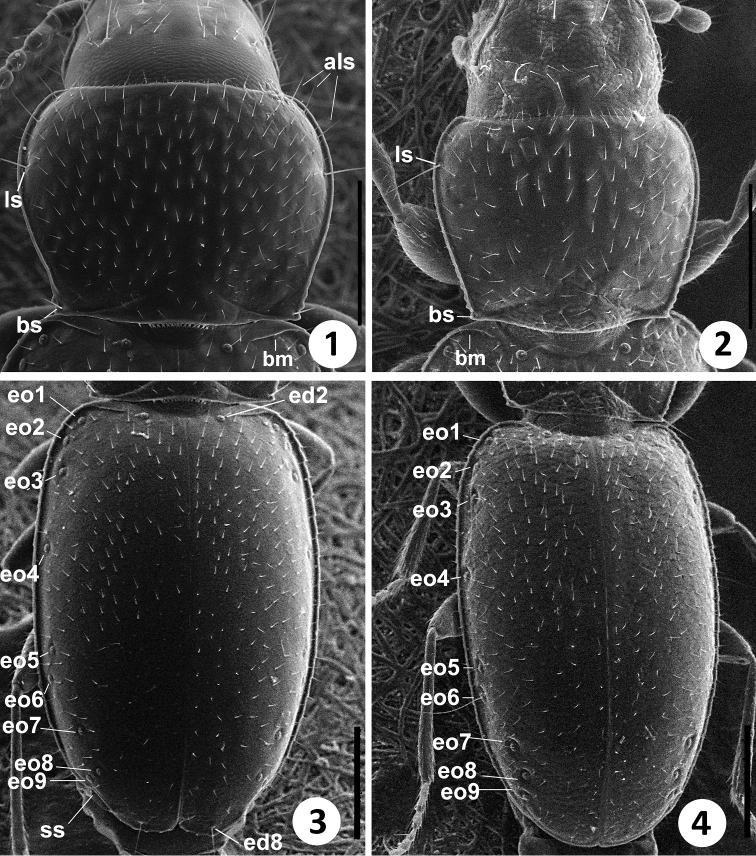
SEM images of body parts of *Zapotecanillus* and *Geocharidius*. Pronotum: **1** *Zapotecanillus oaxacanus* **2** *Geocharidius integripennis*. Elytra: **3** *Zapotecanillus oaxacanus*; **4** *Geocharidius integripennis*. als – anterior lateral pronotal setae; ls – midlateral pronotal seta; bm – basal margin; bs – basilateral pronotal seta; ed2 – scutellar seta; ed8 – apical seta; eo1-9 – setae 1–9 from the umbilicate series; ss – subapical sinuation. Scale bar= 0.2mm.

**Figures 5–8.**
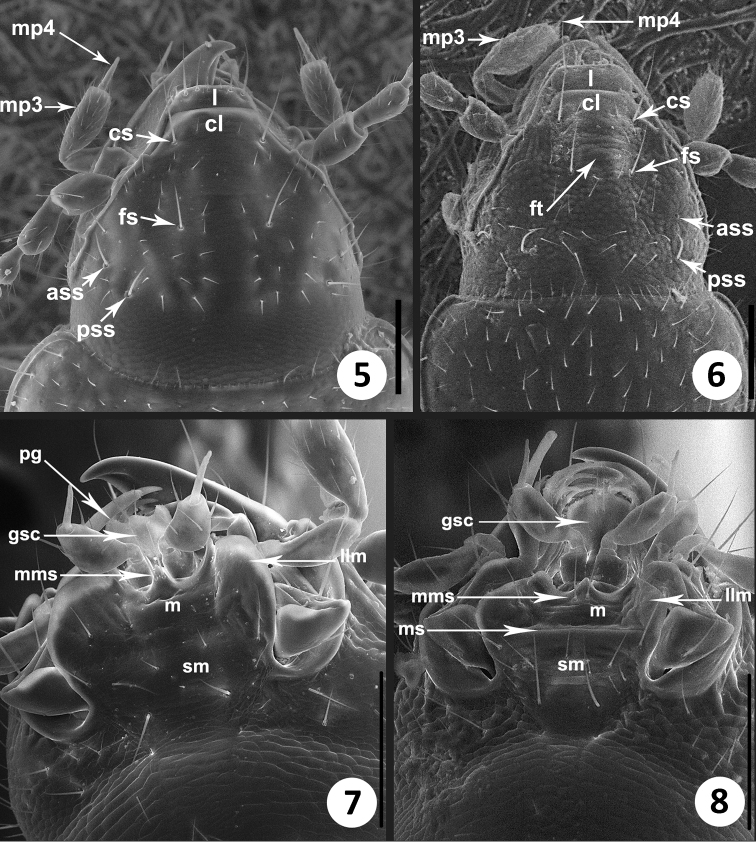
SEM images of structural features of *Zapotecanillus* and *Geocharidius*. Head, dorsal aspect. **5** *Zapotecanillus oaxacanus* **6** *Geocharidius integripennis*. Labium, ventral aspect **7** *Zapotecanillus oaxacanus* **8** *Geocharidius integripennis*. ass – anterior supraorbital seta; cl – clypeus; cs – clypeal seta; fs – frontal seta; ft – frontal tubercle; gsc – glossal sclerite; l – labrum; llm – lateral lobe of mentum; m – mentum; mms – medial mental setae; mp3 – maxillary palpomere 3; mp4 – maxillary palpomere 4; ms – mental-submental suture; pg – paraglossa; pss – posterior supraorbital seta; sm – submentum. Scale bar = 0.1mm.

The members of this genus are distinguished from the other North and Central American representatives of Anillina by the following combination of characters: frontal area of head flat, without a median tubercle; maxillary palps with palpomere 4 longer than 1/3 of palpomere 3; labium with glossal sclerite with short but distinct paraglossae, and with mentum and submentum fused, without mental-submental suture; pronotum forward of the lateral seta and towards anterior angles with a row of elongate setae; elytra without fixed discal setae and with 8^th^ and 9^th^ pores of umbilicate series much closer to each other than the 7^th^ pore is to the 8^th^ (i.e. the “geminate” condition). The most distinctive features of the representatives of the new genus, easily distinguishing them from the species of *Geocharidius*, are the presence of elongate setae at the anterior angles of pronotum forward of the lateral pronotal setae (cf. Fig. 1 versus Fig. 2), a longer maxillary palpomere 4 (cf. Fig. 5 versus Fig. 6), the absence of suture between the mentum and the submentum (cf. Fig. 7 versus Fig. 8), the “geminate” state of the 8^th^ and 9^th^ pores of umbilicate series (cf. Fig. 3 versus Fig. 4), the cross-shaped metendoventrite and the truncate intercoxal process between the hind legs (cf. Fig. 13 versus Fig. 14).

#### Description.

*Size*. SBL range 1.01–1.55 mm.

*Habitus*. Body form weakly to moderately convex, ovoid or subparallel (Figs 24–31).

*Color*. Body bicolored (Fig. 24) or monocolorous (Figs 25–31), brunneorufous, rufotestaceous or testaceous, appendages testaceous.

*Microsculpture*. Dorsal microsculpture of polygonal sculpticells on head, pronotum and elytra. Mesh pattern varies on different body parts. On head, sculpticells transverse, 2-3 times wider than long (Fig. 5). On pronotum, sculpticells longer, 1.5–2 times wider than long. On elytra, sculpticells form mostly isodiametric mesh pattern. Development of microsculpture on pronotum varied among different species.

*Luster*. Body surface shiny.

*Macrosculpture*. Body surface sparsely and finely punctate.

*Vestiture*. Body surface covered with sparse yellow setae of moderate length. Anterior angles of pronotum bear several long setae laterally, which are two times longer than adjacent vestiture (Fig. 1, als).

*Fixed setae*. Primary head setae include a pair of clypeal (cs), a pair of frontal (fs) and two pairs of supraorbital (ass and pss) setae (Fig. 5). Mentum with three pairs of long primary (medial, paramedial and lateral) setae (Fig. 11, mms, pms, lms). Medial mental setae located on mental tooth, not near its base on mentum (Fig. 7, mms). Submentum with two pairs of long primary setae in two rows (lss1, prss) and 1 additional pair of shorter setae (lss2) located laterally (Fig. 11). Maxilla with long stipetal and palpiferal setae (Fig. 12). Pronotum with two long primary lateral setae (middle, ls, and basal, bs) on each side (Fig. 1). Elytra lack discal setae (Fig. 3), but with scutellar (ed2) and apical (ed8) setae. Last two (8^th^ and 9^th^) pores (eo8 and eo9) of umbilicate series much closer to each other than 7^th^ (eo7) pore is to 8^th^ (Fig. 3). Fifth visible sternite of male with two and of female with four setae along the posterior margin.

**Figures 9–12.**
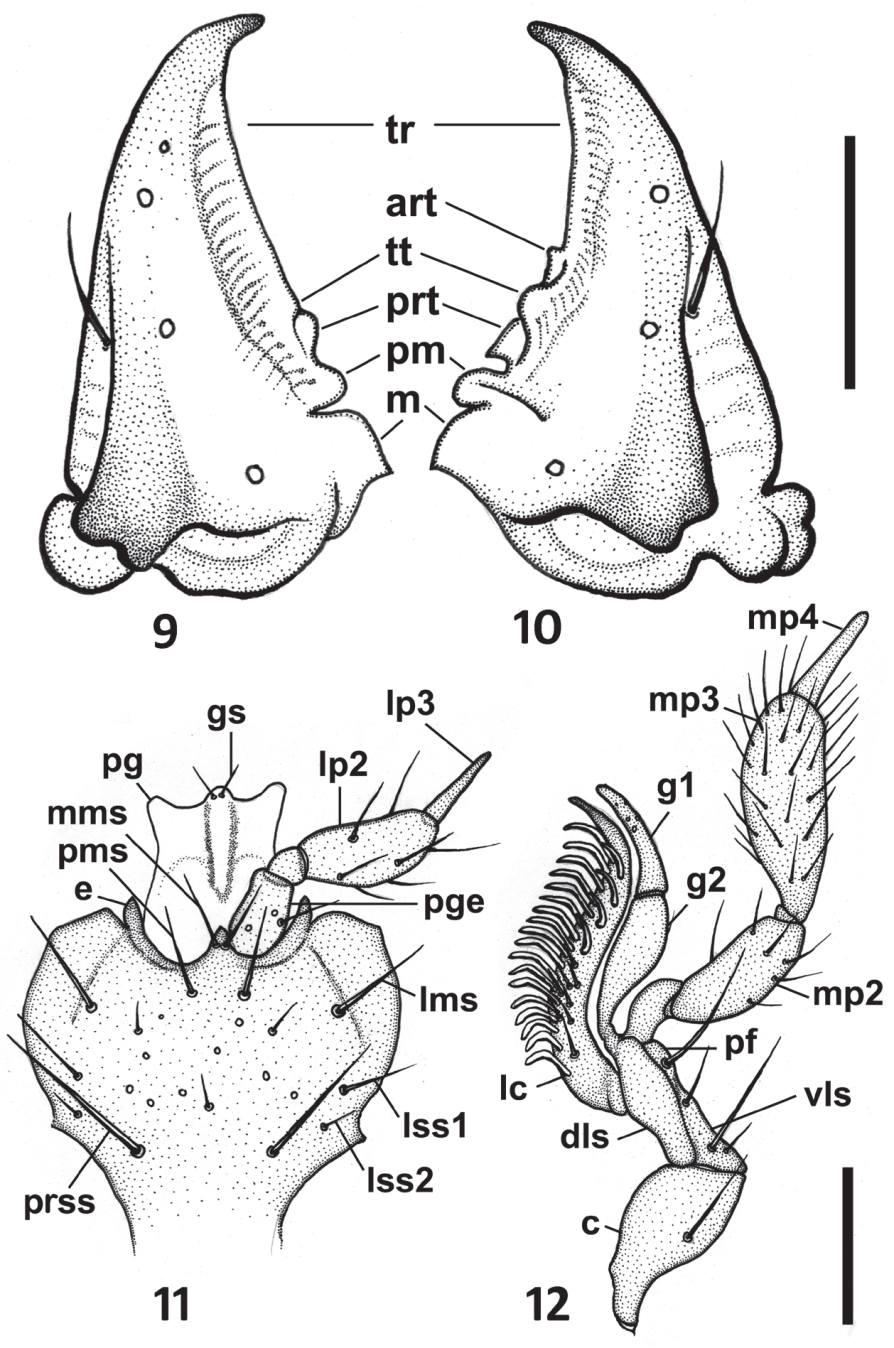
Drawings of the mouthparts of *Zapotecanillus oaxacanus*. **9** left mandible, dorsal aspect **10** right mandible, dorsal aspect **11** labium, ventral aspect **12** right maxilla, ventral aspect. art – anterior retinacular tooth; c – cardo; dls – dorsal lobe of stipes; e – epilobe of mentum; gs – glossal seta; g1 – galeomere 1; g2 – galeomere 2; lc – lacinia; lms – lateral mental seta; lp2 – labial palpomere 2; lp3 – labial palpomere 3; lss1 – lateral submental seta 1; lss2 – lateral submental seta 2; m – molar tooth; mms – medial mental seta; mp2 – maxillary palpomere 2; mp3 – maxillary palpomere 3; mp4 – maxillary palpomere 4; pf – palpifer; pg – paraglossa; pge – palpiger; pm – premolar tooth; pms – paramedial mental seta; prss – primary submental seta; prt – posterior retinacular tooth; tr – terebral ridge; tt – terebral tooth; vls – ventral lobe of stipes. Scale bars = 0.1mm (Figs **9–10**); 0.05mm (Figs **11–12**).

*Head*. Anterior margin of clypeus (cl) straight (Fig. 5). Frontal area flat without tubercle (ft) medially near frontoclypeal suture. Fronto-lateral carinae distinct and long.

*Eyes*. Eyes absent.

*Antennae*. Submoniliform, 11-segmented, extended to about posterior margin of pronotum. Antennomeres 1 and 2 elongate, of equal length and 1.4–1.5 times longer than antennomere 3, which is only slightly elongate and 1.1–1.2 times longer than antennomere 4. Antennomeres 4 to 10 globose, last antennomere (11) conical and 1.6-1.8 times longer than penultimate antennomere.

*Labrum*. Labrum (l) transverse with straight, entire anterior margin with six setae apically, increasing in size from the central pair outwards (Fig. 5).

*Mandibles*. General plan of *Bembidion* type (Maddison 1993). Right mandible with distinct anterior (art) and posterior retinacular (prt), terebral (tt), premolar (pm) and molar (m) teeth (Fig. 10). Left mandible with distinct terebral (tt), posterior retinacular (prt), premolar (pm) and molar (m) teeth only (Fig. 9).

*Maxillae*. Maxillary palps (Fig. 12) similar to *Bembidion* (Maddison 1993) with basal trianguloid cardo, and stipes with dorsal and ventral lobes (dls, vls), dimerous galea (g1, g2), and standard lacinia (lc), with subulate palpomere 4 (mp4). Palpus (Fig. 5) with long 4^th^ palpomere (mp4), 0.4–0.5 length of palpomere 3 (mp3).

*Labium*. Labium (Fig. 7) with mental tooth; mentum and submentum fused, without mental-submental suture (ms) and with moderately enlarged lateral mental lobes, which are translucent along the lateral margins (llm). Glossal sclerite (gsc) with short but distinct paraglossae (pg) laterally and with two setae apically. Central area of mental-submental complex with a field of pores and 1-2 pairs of shorter setae additionally (Fig. 11).

*Prothorax*. Pronotum cordiform, moderately convex, not sinuate (Figs 32–33) or slightly sinuate posteriorly (Figs 34–35). Basal margin of pronotum either straight (Fig. 32), or oblique laterally (Figs 33–34), in one species bisinuate laterally, at posterior angles (Fig. 35). Anterior angles indistinct, broadly rounded. Posterior angles denticulate, without or with 1-2 small denticles in front of the angles. Widths across anterior margin and between posterior angles of approximately equal length (WPa/WPp varies from 0.96 to 1.04 among species).

*Scutellum*. Externally visible, triangular, with narrowly rounded apex.

*Elytra*. Elytra of moderate length (LE/SBL from 0.57 to 0.58 among species) without visible interneurs (Fig. 3). Humeri rounded to form right angle with longitudinal axis of body. Basal margination (bm) distinct and long, reaches half the distance between humeral angle and scutellar pore (Fig. 1). Apical half of elytra with shallow but evident subapical sinuation (ss) (Fig. 3).

*Hind wings*. Absent.

**Figures 13–14.**
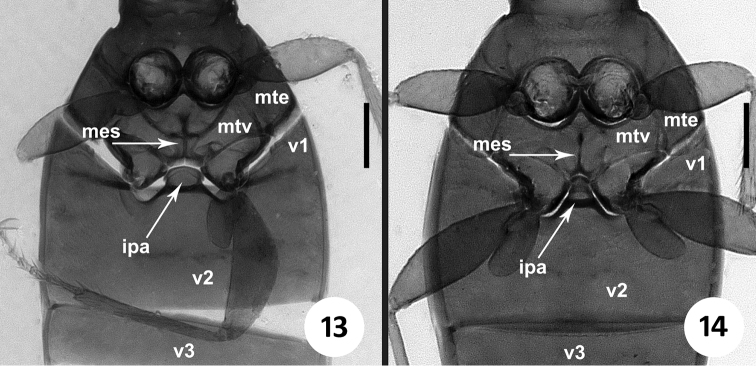
Images of metaventrite and first abdominal ventrites of *Zapotecanillus* and *Geocharidius*. **13** *Zapotecanillus oaxacanus* **14** *Geocharidius integripennis*. ipa – intercoxal process of abdominal ventrite 2; mes – metendosternite; mte – metanepisternum; mtv – metaventrite; v1-v3 – abdominal ventrites 1–3. Scale bar = 0.1mm.

*Pterothorax* (Fig. 13). Metaventrite (mtv) short, distance between meso- and metacoxae about of the diameter of mesocoxa. Metanepisternum (mte) short, subquadrate, with anterior and outer margins of equal length. Metendoventrite (mes) cross-shaped with long lateral arms.

**Figures 15–23.**
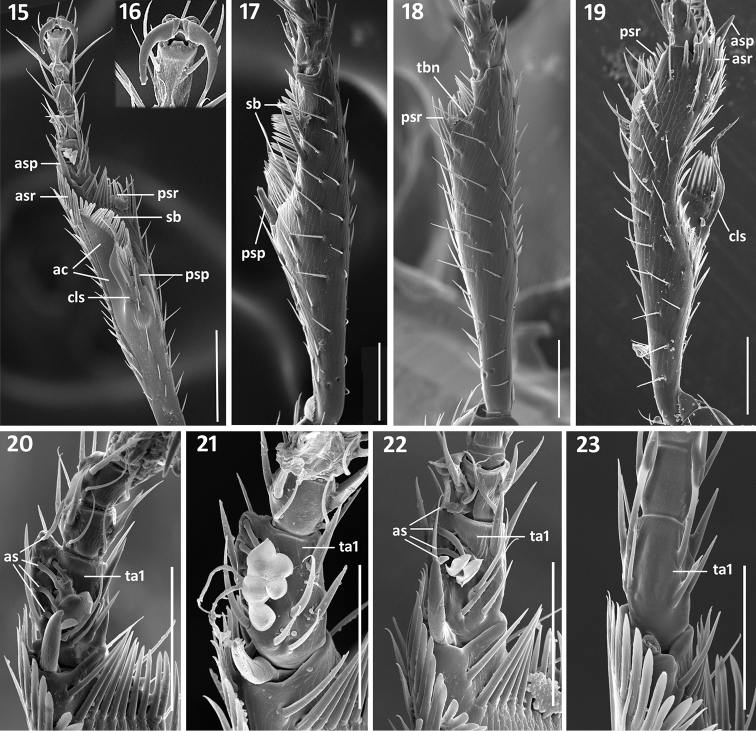
Structural features of front legs of *Zapotecanillus*. Left protibia of *Zapotecanillus oaxacanus*: **15** ventral aspect **16** tarsal claws **17** lateral aspect **18** dorso-lateral aspect. Left protibia of *Zapotecanillus nanus*: **19** dorsal aspect. Male left protarsi, ventral aspect: **20** *Zapotecanillus nanus* **21** *Zapotecanillus iviei* **22** *Zapotecanillus oaxacanus* **23** *Zapotecanillus kavanaughi*. ac – antenna cleaner; as – articulo-seta, asp – anterior spur; asr – anterior setal row; cls – clip setae; psp – posterior spur; psr – posterior setal row; sb – setal band; ta1 – tarsomere 1; tbn – tibial notch. Scale bars = 0.1mm (Figs **15, 17–19**); 0.05mm (Figs **20–23**).

*Legs*. Legs of moderate length, not elongate. Prothoracic legs of males variable in structure of tarsomere 1. Typically, 1^st^ protarsomere markedly dilated apico-laterally with two rows of oval articulo-setae (Stork 1980) on the ventral surface (Figs 20–21). Some species with 1^st^ tarsomere only slightly dilated and with only one (outer) row of oblong articulo-setae (Fig. 22), and other species with 1^st^ tarsomere non-dilated and without adhesive vestiture (Fig. 23). Protibiae (Figs 15, 17–19) with antenna cleaner of type B (Hlavac 1971), with both anterior (asr) and posterior (psr) apical setal rows and concave apico-lateral notch (Fig. 18, tbn). Profemora moderately swollen. Mesotibiae with two terminal spurs and tibial brush. Metafemora unmodified, metatibiae with two terminal spurs. Tarsi pentamerous, 5th and 1^st^ tarsomeres are the longest, 2-4 tarsomeres of equal length on the tarsi of all legs, 1^st^ tarsomere shorter than combined length of 2–4 tarsomeres (Fig. 15). Tarsal claws simple, untoothed (Fig. 16).

**Figures 24–31.**
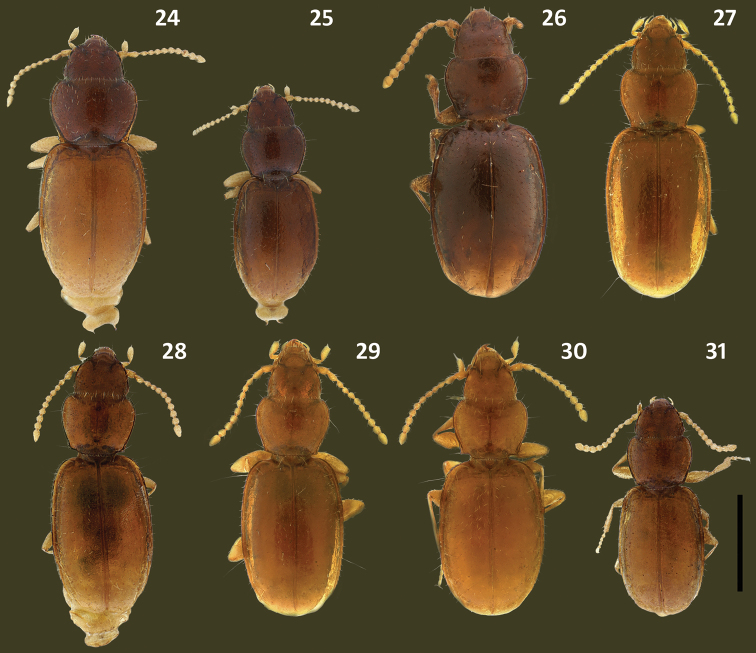
Habitus images of *Zapotecanillus* species. **24** *Zapotecanillus oaxacanus* (MEXICO, Oaxaca, 18.7mi N Valle National), paratype **25** *Zapotecanillus nanus* (MEXICO, Oaxaca, 18.7mi N Valle National), paratype **26** *Zapotecanillus ixtlanus* (MEXICO, Oaxaca, 37mi S Valle National), paratype **27** *Zapotecanillus iviei* (MEXICO, Oaxaca, 2mi S Cerro Pelon), paratype **28** *Zapotecanillus kavanaughi* (MEXICO, Oaxaca, 14km N San Juan), paratype **29** *Zapotecanillus montanus* (MEXICO, Oaxaca, 52mi N Oaxaca), paratype **30** *Zapotecanillus pecki* (MEXICO, Oaxaca, 3.5mi S Suchixtepec), paratype **31** *Zapotecanillus longinoi* (MEXICO, Chiapas, Sierra Morena), paratype. Scale bar = 0.5mm.

**Figures 32–35.**
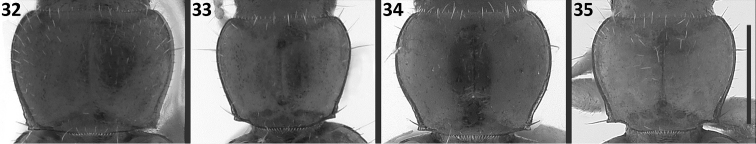
Pronotum images of *Zapotecanillus* species. **32** *Zapotecanillus oaxacanus* (MEXICO, Oaxaca, 18.7mi N Valle National) **33** *Zapotecanillus kavanaughi* (MEXICO, Oaxaca, 14km N San Juan) **34** *Zapotecanillus iviei* (MEXICO, Oaxaca, 2mi S Cerro Pelon) **35** *Zapotecanillus pecki* (MEXICO, Oaxaca, 3.5mi S Suchixtepec). Scale bar = 0.25mm.

*Abdominal ventrites*. Five visible abdominal ventrites: 2^nd^ ventrite longest (Fig. 13), more than 3 times longer than 3^rd^ or 4^th^, 3^rd^ and 4^th^ equal in length; the last, 5^th^, approximately 1.5 times longer than 4^th^. Intercoxal process (ipa) of 2^nd^ ventrite broad, truncate anteriorly (Fig. 13).

*Male genitalia* (Figs 36–59). Median lobe of aedeagus anopic, elongate, twisted and slightly arcuate. Internal sac with two groups of copulatory sclerites: dorsal group represented by 2 plates, and ventral group represented by weakly sclerotized fold or folds. Dorsal plate 1 (dp1) in form of an elongate plate, rounded or pointed at basal end, and tapered into a needle-like structure apically. Dorsal plate 2 (dp2) much smaller than plate 1, also needle-attenuated apically, curved and enlarged towards base; coplanarly adjoined to dorsal plate 1 apically in lateral view and divergent from plate 1 basally as a ventrally directed protuberance; can be seen as a separate structure in some species (Figs 54, 57). Ventral sclerites (vsc) of varied shape, dependent on development of sclerotization. Additional spines or scaled membranous fields of internal sac are absent. Parameres typically bisetose, except right paramere of *Zapotecanillus pecki* 3-setose (Fig. 53). Left paramere large and broad, either evenly tapered to apex (Figs 43, 46) or with short attenuation before setal attachment (Figs 37, 40). Ring sclerite broadly ovate with transverse handle-like extension of varied length and shape (Figs 60–67, hd).

**Figures 36–47.**
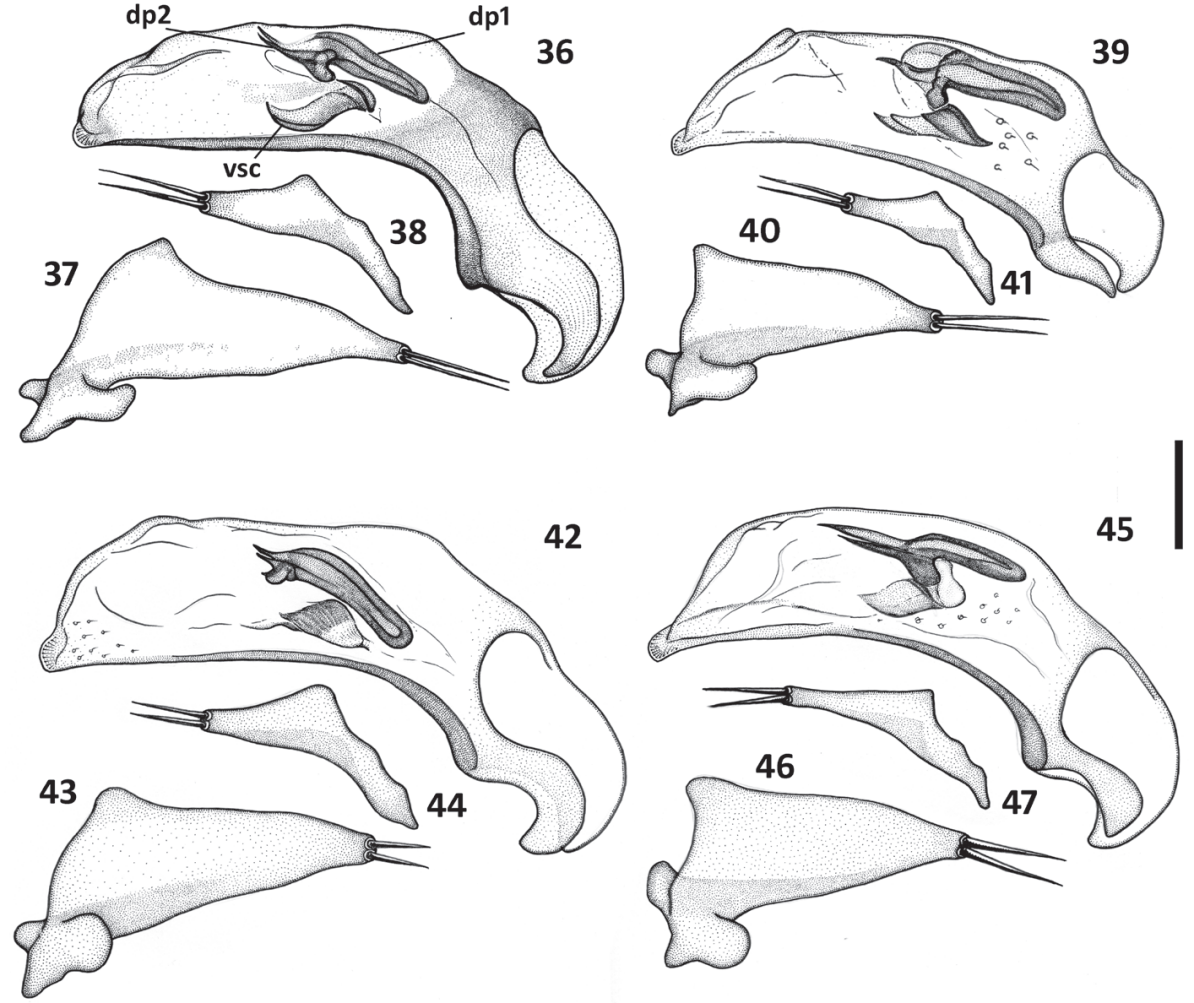
Illustrations of male aedeagus of *Zapotecanillus* species. *Zapotecanillus oaxacanus* (MEXICO, Oaxaca, 18.7mi N Valle National) **36** median lobe, right lateral aspect **37** left paramere, left lateral aspect **38** right paramere, right lateral aspect. *Zapotecanillus nanus* (MEXICO, Oaxaca, 18.7mi N Valle National) **39** median lobe, right lateral aspect **40** left paramere, left lateral aspect **41** right paramere, right lateral aspect. *Zapotecanillus ixtlanus* (MEXICO, Oaxaca, 37mi S Valle National) **42** median lobe, right lateral aspect **43** left paramere, left lateral aspect **44** right paramere, right lateral aspect. *Zapotecanillus iviei* (MEXICO, Oaxaca, 2mi S Cerro Pelon) **45** median lobe, right lateral aspect **46** left paramere, left lateral aspect **47** right paramere, right lateral aspect. dp1 – dorsal plate 1; dp2 – dorsal plate 2; vsc – ventral sclerite. Scale bar = 0.05mm.

**Figures 48–59.**
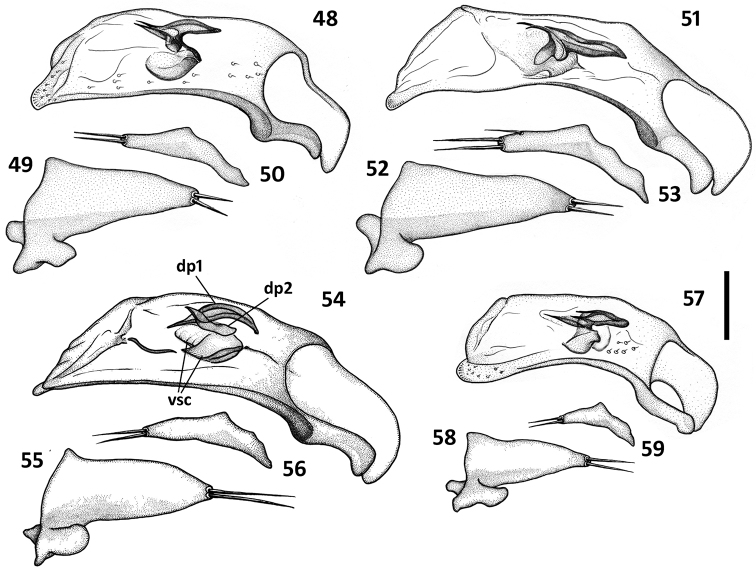
Illustrations of male aedeagus of *Zapotecanillus* species. *Zapotecanillus montanus* (MEXICO, Oaxaca, 52mi N Oaxaca) **48** median lobe, right lateral aspect **49** left paramere, left lateral aspect **50** right paramere, right lateral aspect. *Zapotecanillus pecki* (MEXICO, Oaxaca, 3.5mi S Suchixtepec) **51** median lobe, right lateral aspect **52** left paramere, left lateral aspect **53** right paramere, right lateral aspect. *Zapotecanillus kavanaughi* (MEXICO, Oaxaca, 14km N San Juan) **54** median lobe, right lateral aspect **55** left paramere, left lateral aspect **56** right paramere, right lateral aspect. *Zapotecanillus longinoi* (MEXICO, Chiapas, Sierra Morena) **57** median lobe, right lateral aspect **58** left paramere, left lateral aspect **59** right paramere, right lateral aspect. dp1 – dorsal plate 1; dp2 – dorsal plate 2; vsc – ventral sclerites. Scale bar = 0.05mm.

**Figures 60–67.**
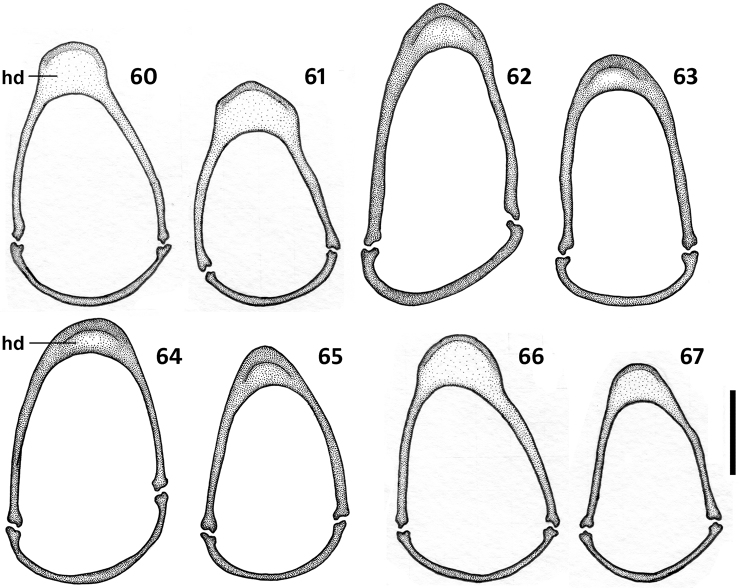
Illustrations of ring sclerite of *Zapotecanillus* species, male genitalia, dorsal aspect. **60** *Zapotecanillus oaxacanus* (MEXICO, Oaxaca, 18.7mi N Valle National) **61** *Zapotecanillus nanus* (MEXICO, Oaxaca, 18.7mi N Valle National) **62** *Zapotecanillus ixtlanus* (MEXICO, Oaxaca, 37mi S Valle National) **63** *Zapotecanillus iviei* (MEXICO, Oaxaca, 2mi S Cerro Pelon) **64** *Zapotecanillus pecki* (MEXICO, Oaxaca, 3.5mi S Suchixtepec) **65** *Zapotecanillus montanus* (MEXICO, Oaxaca, 52mi N Oaxaca) **66** *Zapotecanillus kavanaughi* (MEXICO, Oaxaca, 14km N San Juan) **67** *Zapotecanillus longinoi* (MEXICO, Chiapas, Sierra Morena). hd – handle of ring sclerite. Scale bar = 0.1mm.

**Figures 68–71.**
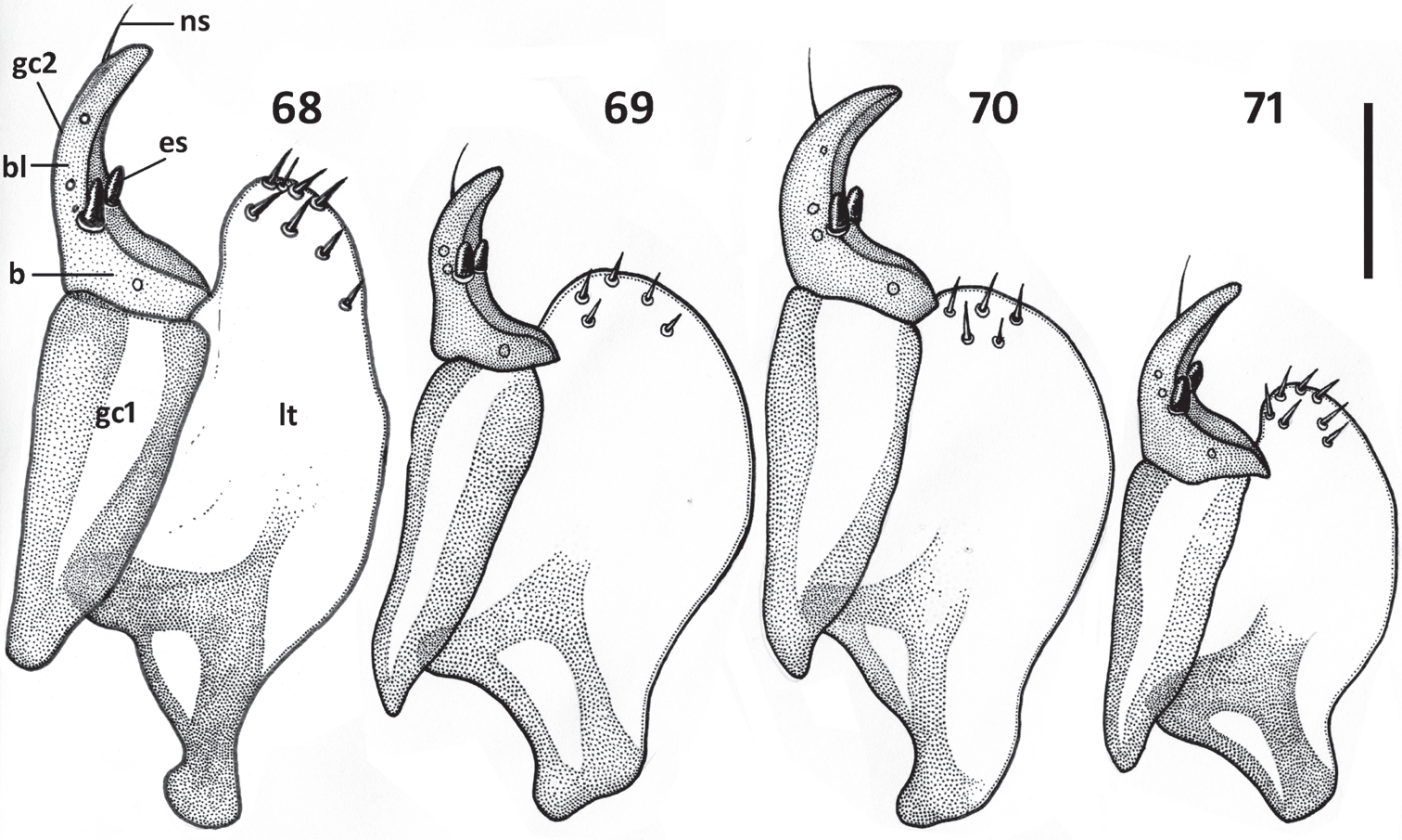
Illustrations of ovipositor sclerites of *Zapotecanillus* species. **68** *Zapotecanillus oaxacanus* (MEXICO, Oaxaca, 18.7mi N Valle National) **69** *Zapotecanillus nanus* (MEXICO, Oaxaca, 15.1mi N Valle National) **70** *Zapotecanillus kavanaughi* (MEXICO, Oaxaca, 14km N San Juan) **71** *Zapotecanillus longinoi* (MEXICO, Chiapas, Sierra Morena). b – base of gonocoxite 2, bl – blade of gonocoxite 2, es – ensiform seta; gc1 – gonocoxite 1; gc 2 – gonocoxite 2; lt – laterotergite; ns – nematiform seta. Scale bar = 0.05mm.

**Figures 72–76.**
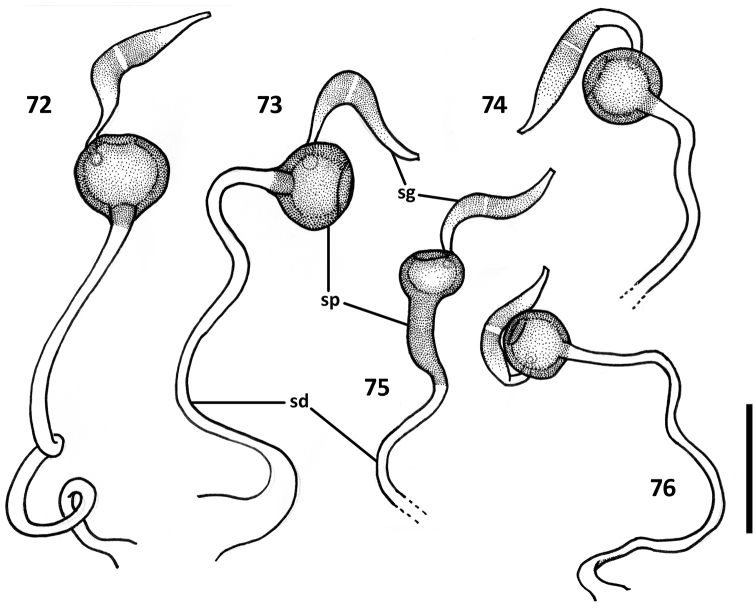
Illustrations of spermatheca, spermathecal duct, and spermathecal gland of *Zapotecanillus* species. **72** *Zapotecanillus oaxacanus* (MEXICO, Oaxaca, 18.7mi N Valle National) **73** *Zapotecanillus nanus* (MEXICO, Oaxaca, 15.1mi N Valle National) **74** *Zapotecanillus iviei* (MEXICO, Oaxaca, 2mi S Cerro Pelon) **75** *Zapotecanillus kavanaughi* (MEXICO, Oaxaca, 14km N San Juan) **76** *Zapotecanillus longinoi* (MEXICO, Chiapas, Sierra Morena). sd – spermathecal duct; sg – spermathecal gland; sp – spermatheca. Scale bar = 0.05mm.

*Female genitalia* (Figs 68–76). Ovipositor sclerites: Gonocoxite 1 asetose (gc1). Gonocoxite 2 triangular (gc2), 1.6–1.8 times longer than its basal width, slightly to moderately curved, with 2 lateral ensiform (es) and apical nematiform (ns) setae. Laterotergite (lt) with 5–8 setae. Internal genitalia with spermatheca sclerotized, rufous, spherical and ball-shaped in most species, fusiform with a bulb-like enlargement apically in *Zapotecanillus kavanaughi* (Fig. 75).

#### Included taxa.

The new genus includes eight species: *Zapotecanillus oaxacanus* sp. n., *Zapotecanillus nanus* sp. n., *Zapotecanillus ixtlanus* sp. n., *Zapotecanillus iviei* sp. n., *Zapotecanillus montanus* sp. n., *Zapotecanillus pecki* sp. n., *Zapotecanillus kavanaughi* sp. n., and *Zapotecanillus longinoi* sp. n.

#### Geographical distribution.

**Figure 77.**
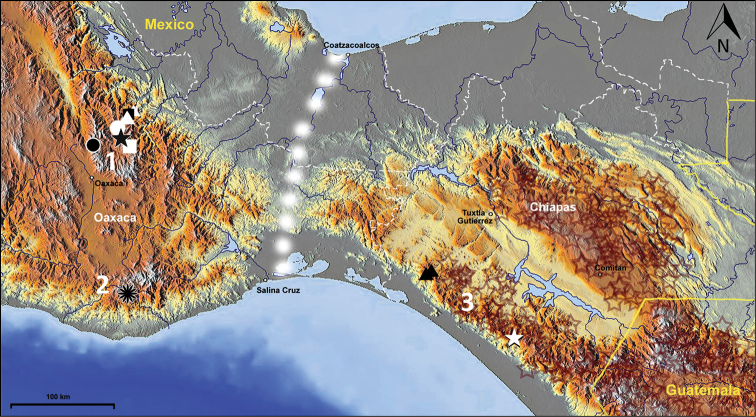
Map of southern Mexico and adjacent part of Guatemala, showing positions of locality records for the species of *Zapotecanillus*. *Zapotecanillus oaxacanus* – black quadrangle; *Zapotecanillus nanus* – white triangles; *Zapotecanillus ixtlanus* – white circle; *Zapotecanillus iviei* – black star; *Zapotecanillus kavanaughi* – black circle; *Zapotecanillus montanus* – white quadrangle; *Zapotecanillus pecki* – black flower; *Zapotecanillus longinoi* – black triangles; *Zapotecanillus* sp. – white star. Brown stars – range of *Geocharidius* species (original data). 1 – Sierra Madre de Oaxaca; 2 – Sierra Madre del Sur; 3 – Sierra Madre de Chiapas. White dots – the Isthmus of Tehuantepec.

The species of this genus are known from three mountain ranges of Mexico: the Sierra Madre de Oaxaca, the Sierra Madre del Sur and the Sierra Madre de Chiapas, within the states of Oaxaca and Chiapas (Fig. 77). This type of distribution best fits Halffter’s (1987) Meso-American Montane Distribution Pattern.

#### Way of life.

According to the label information, specimens of the new genus were taken from the leaf litter within the 1200–3000m range of altitudes at the Sierra Madre de Oaxaca, and in mesophyll and cloud forests within the 1330–2140m range of altitudes at the Sierra Madre de Chiapas. Collecting dates are May, June, July and August. One specimen from the Sierra Madre de Oaxaca was taken “under bark hardwood”.

#### Relationships.

The position of *Zapotecanillus* within the North and Central American Anillina is unclear at present, and awaits molecular data analysis or further discoveries and subsequent morphological analyses of the Middle American anilline taxa. Members of this new genus differ principally from those of the southern stock of Middle American anilline genera (*Geocharidius* Jeannel, *Honduranillus* Zaballos, *Mexanillus* Vigna Taglianti) in having a different arrangement of the last three pores of the umbilicate series and the fused labium, and from geographically proximate *Geocharidius* and *Mexanillus* in having distinct paraglossae. They differ from members of the northern stock of North American anilline genera (*Anillaspis* Casey, *Anillinus* Casey, *Anillodes* Jeannel, *Micranillodes* Jeannel, *Serranillus* Barr) in lacking fixed discal pores on the elytra.

The key provided below allows distinguishing members of the new genus from those of the other continental North and Middle American anilline genera:

**Table:** 

1	Elytra without fixed discal setae (Figs 3–4). Mexico and Central America	2
–	Elytra with 3 pairs of fixed discal setae. North of Mexico	other genera (*Anillaspis* Casey, *Anillinus* Casey, *Anillodes* Jeannel, *Micranillodes* Jeannel, *Serranillus* Barr)
2	Labium fused, without mental-submental suture (Fig. 7). Pores 8 and 9 of umbilicate series geminate, much closer to each other, than 8^th^ to 7^th^ (Fig. 3)	*Zapotecanillus*,gen. n.
–	Labium free, with distinct mental-submental suture (Fig. 8). Pores 7, 8 and 9 of umbilicate series equidistant (Fig. 4)	3
3	Pronotum convex. Glossal sclerite without distinct paraglossae (Fig. 8). Size smaller, less than 2.7mm	4
–	Pronotum subdepressed. Glossal sclerite with distinct paraglossae (as in Fig. 7). Size larger, 3mm	*Honduranillus* Zaballos
4	Length greater than 2.2 mm. Head with impressed, subparallel frontal furrows and shortened latero-frontal carinae. Appendages, especially tarsi, elongate; 1^st^ tarsomeres of middle and hind legs very long, longer than the length of tarsomeres 2-4 combined. Troglobitic beetles	*Mexanillus* Vigna Taglianti
–	Length less than 1.9 mm. Head with faint and divergent frontal furrows and long latero-frontal carinae. Appendages of standard length, 1^st^ tarsomere of middle and hind legs shorter than tarsomeres 2-4 combined. Litter-dwelling beetles	*Geocharidius* Jeannel

### Zapotecanillus oaxacanus sp. n.

http://zoobank.org/86234081-8CB0-45D0-861D-721A0C05174A

http://species-id.net/wiki/Zapotecanillus_oaxacanus

Figs 1 , 3 , 5 , 7 , 9 –13 , 15–18 , 22 , 24 , 32 , 36–38 , 60 , 68 , 72 , 77 , 90–92 , 94

#### Type material.

HOLOTYPE, male, in NMNH, point-mounted, labeled: \MEXICO. Oaxaca 18.7mi S Valle Nacional 5200’ 17 Aug.1973\ A.Newton Collector \ Loan from NMNH 2051867\. PARATYPES (8 ex., 4♂2♀ were dissected), labeled same as a holotype, except two specimens, which have an additional label: \*Geocharidius* *n. sp.* det. T.L.Erwin *76*\ each, where italicized font means handwritten (deposited in CAS, NMNH).

#### Specific epithet.

The specific epithet is a Latinized adjective in the masculine form based on Oaxaca, the state of Mexico from which the new species is described.

#### Type locality.

Mexico, Oaxaca, 18.7mi S from Valle Nacional.

#### Recognition.

Adults of this new species can be distinguished easily from those of other species of the genus by the following combination of external characters: bicolored and robust appearance, comparatively small head and distinctly transverse pronotum.

#### Description.

*Size*. Medium-sized for the genus (SBL range 1.30-1.36 mm, mean 1.33±0.049 mm, n=8).

*Habitus*. Body form (Fig. 24) moderately convex, slightly elongate (WE/SBL 0.41±0.09), head narrow for genus compared to pronotum (WH/WPm 0.69±0.015), pronotum wide compared to elytra (WPm/WE 0.81±0.017).

*Color*. Body bicolored: head and pronotum brunneorufous, elytra rufotestaceous, appendages testaceous.

*Microsculpture*. Disc of pronotum with well-developed microsculpture.

*Prothorax*. Pronotum (Fig. 32) relatively long (LP/LE 0.44±0.006) and markedly transverse (WPm/LP 1.33±0.022), with lateral margins straight and moderately constricted posteriorly (WPm/WPp 1.32±0.034). Basal margin straight. Contour of posterior angles nearly rectangular (100–110°) with 1–2 small denticles in front of the angles.

*Elytra* (Fig. 3). Convex, not depressed along suture, comparatively wide (WE/LE 0.73±0.013). Margins rounded, slightly divergent in basal half, evenly rounded to apex in apical half, maximal width of elytra at midpoint.

*Legs*. 1^st^ male protarsomere only slightly dilated (Fig. 22).

*Male genitalia*. Median lobe of aedeagus (Fig. 36), with short and transverse apex, broadly rounded at tip. Dorsal plate 1 long, with apical pointed attenuation of moderate length. Dorsal plate 2 joined to plate 1 at its middle ventrally, where it forms a distinct protuberance. Ventral sclerites elongate, with subparallel sides and obliquely stretched from dorsal plates towards ventral margin of median lobe. Right paramere short and moderately wide (Fig. 38). Left paramere with distinct apical constriction (Fig. 37). Ring sclerite with long handle-like extension, widely rounded apically (Fig. 60).

*Female genitalia*. Gonocoxite 2 rather long, with slightly curved blade and rounded apex (Fig. 68). Laterotergite with 7-8 setae. Spermatheca standard for genus (Fig. 72).

**Geographical distribution.** This species is known only from the type locality in the Sierra de Juárez Range, a part of the Sierra Madre de Oaxaca, within the high course of the Rio de Valle Nacional (Figs 77 and 94, black quadrangles).

**Way of life.** Specimens of this species were collected at an altitude of 5200 feet (1600 m).

**Relationships.** The armature of internal sac of *Zapotecanillus oaxacanus* males is nearly indistinguishable from that of *Zapotecanillus nanus* and *Zapotecanillus ixtlanus* males, described below, clearly suggesting both of them as closest relatives. The former species is sympatric with *Zapotecanillus oaxacanus* and, based on the same label data, may also be syntopic (i.e., their members may occur together in the same habitat). See also Fig. 90 for cladistic affinities.

### Zapotecanillus nanus sp. n.

http://zoobank.org/E3677571-83C4-4836-9252-BC1B5C11129E

http://species-id.net/wiki/Zapotecanillus_nanus

Figs 19 , 20 , 25 , 39–41 , 61 , 69 , 73 , 77 , 90–92 , 94

#### Type material.

HOLOTYPE, male, in NMNH, point-mounted, labeled: \ MEX. Oax., 15mi. S. Valle Nacional, 4000’ 21.V.1971 S.Peck Ber.204, leaf litter \ Borrowed Ex. J.M. Campbell \ USNMNH 2051867\. PARATYPES (27 ex., 4♂4♀ were dissected), 4 ex. labeled same as holotype, except one specimen, which has an additional label: \*Geocharidius* *n. sp.* det. T.L.Erwin *76*\, where italicized font means handwriting.; 12 ex. labeled: MEXICO: Oaxaca, 15mi. S. Valle Nacional, 4000’ 21 May 1971, leaf litter, S. Peck \ THOMAS C. BARR COLLECTION 2011 Acc. No. 38014\; 1 ex. labeled: \ MEXICO: Oaxaca 15.1mi S Valle Nacional 4300’ VIII-11-18-1973 \ under bark hardwood A.Newton \ Borrowed ex. MCZ \ Loan from USNMNH 2051867\; 10 ex. labeled: \ MEXICO. Oaxaca 18.7mi S Valle Nacional 5200’ 17 Aug.1973\ A.Newton Collector \ Loan from USNMNH 2051867\ (deposited in CAS, CMNH, NMNH).

#### Specific epithet.

The specific epithet is a Latin adjective, *nanus*, in the masculine form, meaning *dwarf*, *miniature*, and refers to the small size of the beetles.

#### Type locality.

Mexico, Oaxaca, 15mi S from Valle Nacional.

#### Recognition.

Adults of this new species are distinguished from those of other species of the genus by the combination of small size and brunneorufous color; and males can be further distinguished by the shape of the median lobe (Fig. 39).

#### Description.

*Size*. Small for genus (SBL range 1.02–1.16 mm, mean 1.10±0.049 mm, n=12).

*Habitus*. Body form (Fig. 25) moderately convex, slightly elongate (WE/SBL 0.41±0.09), head of normal proportions for genus (WH/WPm 0.75±0.016), pronotum narrow compared to elytra (WPm/WE 0.75±0.028).

*Color*. Body monocolorous, brunneorufous, appendages testaceous.

*Microsculpture*. Microlines partially effaced on disc of pronotum.

*Prothorax*. Pronotum relatively long (LP/LE 0.43±0.020) and moderately transverse (WPm/LP 1.26±0.030), with margins straight and markedly constricted posteriorly (WPm/WPp 1.38±0.044). Basal margin slightly oblique laterally. Posterior angles small, contour of posterior angles obtuse (112–126°) without or with 1 small denticle in front of the angles.

*Elytra*. Slightly convex, not depressed along suture, comparatively wide (WE/LE 0.72±0.025). Margins rounded, moderately divergent in basal half, evenly rounded to apex in apical third, maximal width of elytra slightly behind the midpoint.

*Legs*. 1^st^ male protarsomere markedly dilated apico-laterally (Fig. 20).

*Male genitalia*. Median lobe of aedeagus (Fig. 39), with small, slightly elongated apex, angulately rounded at tip. Dorsal plate 1 long, with apical pointed attenuation of moderate length. Dorsal plate 2 joined to plate 1 at its middle ventrally, where it forms a distinct protuberance. Ventral sclerites with sides divergent ventrally, trianguloid in shape. Right paramere short and moderately narrow (Fig. 41). Left paramere with distinct apical constriction (Fig. 40). Ring sclerite with long handle-like extension, pointed apically (Fig. 61).

*Female genitalia*. Gonocoxite 2 comparatively short, with slightly curved blade and narrowly rounded apex (Fig. 69). Laterotergite with 5-6 setae. Spermatheca standard for genus (Fig. 73).

#### Geographical distribution.

The species is known from a few localities in the Sierra de Juárez Range, a part of the Sierra Madre de Oaxaca, along the ~5km stretch of the Rio de Valle Nacional (Figs 77 and 94, white triangles).

#### Way of life.

According to the label data, the elevations of localities range from 4000’ to 5200’ (1200–1600 m).

#### Relationships.

Aedeagal characters (shape of dorsal plates and left paramere) suggest that *Zapotecanillus nanus* is most closely related to the sympatric *Zapotecanillus oaxacanus*. See also Fig. 90 for cladistic affinities.

### Zapotecanillus ixtlanus sp. n.

http://zoobank.org/3349A2CB-9EA6-4F1E-8472-F9C7C4854CDC

http://species-id.net/wiki/Zapotecanillus_ixtlanus

Figs 26 , 42–44 , 62 , 77 , 90 , 92 , 94

#### Type material.

HOLOTYPE, male, in CMNH, point-mounted, labeled: \MEXICO: Oaxaca, 32 miles S Valle Nacional, 7000 ft. 23 May 1971, ex. leaf litter, Peck\ THOMAS C. BARR COLLECTION 2011 Acc. No. 38014\. PARATYPES (17 ex., 3♂1♀ were dissected), 10 ex. labeled same as a holotype; one male labeled: 8500’, 37mi. S. Valle Nacional, Oax. Mex. V.24.1971 H.Howden \ Borrowed Ex. H.F. Howden \ Loan from USNMNH 2051867\; 3 ex. labeled: \MEXICO: Oaxaca, 37 mi. S Valle Nacional, 8500 ft. 23 May 1971, ex. leaf litter, Peck\ THOMAS C. BARR COLLECTION 2011 Acc. No. 38014\; 3 ex. labeled: \MEXICO: Oaxaca, 37 mi. S Valle Nacional, oak litter, 25 May 1971, S. Peck\ THOMAS C. BARR COLLECTION 2011 Acc. No. 38014\ (deposited in CAS, CMNH, NMNH).

#### Specific epithet.

The specific epithet is a Latinized adjective in the masculine form based on *Ixtlan*, the district of the state of Oaxaca, Mexico from which the new species is described.

#### Type locality.

Mexico, Oaxaca, 32mi. S. Valle Nacional.

#### Recognition.

Males of this new species can be distinguished from those of other species of the genus by the combination of the large size and shape of the median lobe (Fig. 42).

#### Description.

*Size*. Large for genus (SBL range 1.32–1.53 mm, mean 1.39±0.069 mm, n=12).

*Habitus*. Body form (Fig. 26) moderately convex, slightly elongate (WE/SBL 0.41±0.016), head of normal proportions for genus (WH/WPm 0.72±0.013), pronotum narrow compared to elytra (WPm/WE 0.72±0.038).

*Color*. Body monocolorous, rufobrunneous, appendages testaceous.

*Microsculpture*. Disc of pronotum with well-developed microsculpture.

*Prothorax*. Pronotum relatively small (LP/LE 0.39±0.020) and moderately transverse (WPm/LP 1.31±0.039), with margins straight and distinctly constricted posteriorly (WPm/WPp 1.41±0.050). Basal margin oblique laterally. Contour of posterior angles obtuse (111–123°) with a small denticle.

*Elytra*. Convex, not depressed along suture, of moderate width (WE/LE 0.70±0.023). Margins subparallel at middle, slightly divergent in basal third, evenly rounded to apex in apical third, maximal width of elytra slightly behind midpoint.

*Legs*. 1^st^ male protarsomere markedly dilated apico-laterally.

*Male genitalia*. Median lobe of aedeagus (Fig. 42) with short semicircular apex. Dorsal plate 1 long, with short apical attenuation. Dorsal plate 2 joined to plate 1 at its middle ventrally, where it forms a distinct protuberance. Ventral sclerites slightly sclerotized, trianguloid in shape. Right paramere short and moderately wide (Fig. 44). Left paramere without apical constriction (Fig. 43). Ring sclerite with short handle-like extension, pointed apically (Fig. 62).

*Female genitalia*. Spermatheca standard for genus.

#### Geographical distribution.

The species is known only from the type locality in the Sierra Juárez Range, a part of the Sierra Madre de Oaxaca (Figs 77 and 94, white circle).

#### Way of life.

Specimens were collected at altitudes 7000-8500’ (2100–2600 m) in oak litter.

#### Relationships.

Aedeagal characters (shape of the median lobe and dorsal plates) suggest that *Zapotecanillus ixtlanus* is closely related to *Zapotecanillus oaxacanus* and *Zapotecanillus nanus*. See also Fig. 90 for cladistic affinities.

### Zapotecanillus iviei sp. n.

http://zoobank.org/7B74C66E-1EEB-4706-8AFE-A7B6D9A056CC

http://species-id.net/wiki/Zapotecanillus_iviei

Figs 21 , 27 , 34 , 45–47 , 63 , 74 , 77 , 90 , 94

#### Type material.

HOLOTYPE, male, in CAS, point-mounted, labeled: \ MEX: OAXACA, 2 mi S. Cerro Pelon, 8–9000 ft. 03 JUL 1982 M.A. Ivie colr.\ ex rotten pine \ *Geocharidius* n. sp. det. M. A. Ivie 1983 (handwriting)\. PARATYPES (34 ex., 4♂2♀ were dissected), labeled same as a holotype (deposited in CAS, MTEC).

#### Specific epithet.

The specific epithet is a Latinized eponym in the genitive case, and is based on the surname of Michael A. Ivie, Associate Professor and Curator of Entomology at the Montana State University, the collector of the type series of the species.

#### Type locality.

Mexico, Oaxaca, 2 miles S Cerro Pelon.

#### Recognition.

Adults of this new species can be distinguished from those of other species of the genus by the combination of elongate habitus and comparatively narrow pronotum; and males can be further distinguished by the shape of the copulatory sclerites of the median lobe (Fig. 45).

#### Description.

*Size*. Large for genus (SBL range 1.34–1.55 mm, mean 1.43 ± 0.066 mm, n=21).

*Habitus*. Body form (Fig. 27) slightly convex, moderately elongate (WE/SBL 0.39±0.09), head of normal proportions for genus (WH/WPm 0.74±0.013), pronotum narrow compared to elytra (WPm/WE 0.72±0.022).

*Color*. Body monocolorous, rufotestaceous, appendages testaceous.

*Microsculpture*. Partially effaced on disc of pronotum.

*Prothorax*. Pronotum (Fig. 34) relatively short (LP/LE 0.39±0.011) and slightly transverse (WPm/LP 1.22±0.025), with margins slightly sinuate and markedly constricted posteriorly (WPm/WPp 1.42±0.039). Basal margin oblique laterally. Contour of posterior angles obtuse (114–124°) without or with 1 small denticle in front of the angles.

*Elytra*. Slightly convex, not depressed along suture, rather narrow (WE/LE 0.66±0.019). Margins subparallel at middle, slightly divergent in basal forth, evenly rounded to apex in apical forth, maximal width of elytra at midpoint.

*Legs*. 1^st^ male protarsomere markedly dilated apico-laterally (Fig. 21).

*Male genitalia*. Median lobe of aedeagus (Fig. 45), with small, slightly elongated apex, angulately rounded at tip. Dorsal plate 1 long, with long apical attenuation. Dorsal plate 2 joined to plate 1 at its middle ventrally, where it forms a distinct protuberance. Ventral sclerites weakly sclerotized. Right paramere rather long and narrow (Fig. 47). Left paramere without apical constriction (Fig. 46). Ring sclerite with short handle, which is widely rounded apically (Fig. 63).

*Female genitalia*. Spermatheca (Fig. 74) standard for genus.

#### Geographical distribution.

The species is known only from the type locality in the Sierra Juárez Range, a part of the Sierra Madre de Oaxaca (Figs 77 and 94 black star).

#### Way of life.

According to the label data (elevation ranges 2600–2700m), these beetles inhabit the pine-oak forest zone of the Sierra Madre de Oaxaca.

#### Relationships.

Externally, adults of *Zapotecanillus iviei* are similar to those of *Zapotecanillus kavanaughi*, *Zapotecanillus pecki* and *Zapotecanillus montanus*, described below, but the armature of the internal sac of the median lobe suggests closer relatedness to *Zapotecanillus oaxacanus*, *Zapotecanillus nanus* and *Zapotecanillus ixtlanus*. See also Fig. 90 for cladistic affinities.

### Zapotecanillus kavanaughi sp. n.

http://zoobank.org/129C5EFE-EB44-4528-A228-DF4315902D76

http://species-id.net/wiki/Zapotecanillus_kavanaughi

Figs 23 , 28 , 33 , 54–56 , 66 , 70 , 75 , 77 , 90 , 94

#### Type material.

HOLOTYPE, male, in CMNC, point-mounted, labeled: \MEX. Oax. 14km N SanJuan del Estado 2600m. 4-VIII.1986 H. & A. Howden\ berlese\ CMNC\. PARATYPES (8 ex., 2♂2♀ were dissected), labeled same as a holotype (deposited in CAS, CMNC).

#### Specific epithet.

The specific epithet is a Latinized eponym in the genitive case, and is based on the surname of David H. Kavanaugh, Senior Curator of the Entomology Department of the California Academy of Sciences, whose enthusiastic efforts in locating and borrowing the material for the current investigation were so magnanimous and productive.

#### Type locality.

Mexico, Oaxaca, 14 km N San Juan del Estado.

#### Recognition.

Adults of this new species are distinguished from those of other species of the genus by the combination of elongate habitus and comparatively narrow pronotum; and males can be further distinguished by the shape of median lobe (Fig. 54).

#### Description.

*Size*. Medium-sized for genus (SBL range 1.25–1.42 mm, mean 1.34±0.055 mm, n=9).

*Habitus*. Body form (Fig. 28) slightly convex, moderately elongate (WE/SBL 0.38±0.13), head of normal proportions for genus (WH/WPm 0.77±0.014), pronotum narrow compared to elytra (WPm/WE 0.74±0.020).

*Color*. Body monocolorous, rufotestaceous, appendages testaceous.

*Microsculpture*. Partially effaced on disc of pronotum.

*Prothorax*. Pronotum (Fig. 33) relatively short (LP/LE 0.40±0.009) and slightly transverse (WPm/LP 1.23±0.031), with margins rectilinear and distinctly constricted posteriorly (WPm/WPp 1.36±0.039). Basal margin oblique laterally. Contour of posterior angles obtuse (116–122°) with 1–2 small denticles in front of the angles.

*Elytra*. Slightly convex, not depressed along suture, rather narrow (WE/LE 0.66±0.023). Margins almost subparallel, slightly divergent in basal half, evenly rounded to apex in apical third, maximal width of elytra posterior to midpoint.

*Legs*. 1^st^ male protarsomere not dilated, without adhesive vestiture (Fig. 23).

*Male genitalia*. Median lobe of aedeagus (Fig. 54), with very narrow, elongate apex. Dorsal plate 1 short, pointed basally, with apical attenuation of moderate length. Dorsal sclerite 2 in a form of a separate structure, crosses plate 1 at apical third. Ventral sclerites slightly sclerotized. Right paramere rather long and moderately wide (Fig. 56). Left paramere without apical constriction (Fig. 55). Ring sclerite with long handle-like extension, widely rounded apically (Fig. 66).

*Female genitalia*. Gonocoxite 2 comparatively short, with moderately curved blade and rounded apex (Fig. 70). Laterotergite with 5-6 setae. Spermatheca atypical for genus (Fig. 75).

#### Geographical distribution.

The species is known only from the type locality in the Sierra Aloapaneca Range, a part of the Sierra Madre de Oaxaca (Figs 77 and 94, black circle).

#### Way of life.

All beetles were collected at an elevation of 2600 m.

#### Relationships.

Externally, adults of *Zapotecanillus kavanaughi* are similar to those of *Zapotecanillus iviei*, *Zapotecanillus pecki* and *Zapotecanillus montanus*, described below, but males and females differ from those of these species in features of the median lobe and shape of the spermatheca, respectively. See also Fig. 90 for cladistic affinities.

### Zapotecanillus montanus sp. n.

http://zoobank.org/805794A0-A363-4C93-9041-C40DDBB9675D

http://species-id.net/wiki/Zapotecanillus_montanus

Figs 29 , 48–50 , 65 , 77 , 90 , 94

#### Type material.

HOLOTYPE, male, in CMNH, point-mounted, labeled: \ MEXICO, Oaxaca, 52miles N of Oaxaca, Ber.202, sink litter, 17 May 1971 S.B.Peck collector\ CMNH \. PARATYPES (14 ex., 3♂4♀ were dissected), 6 ex. labeled same as a holotype; one female labeled: \ MEX. Oax., 52mi. N Oaxaca, 9500’ 17.V.71 S.Peck Ber.202, leaf lit.\ *Anillinus* (handwriting)\; 7 ex. labeled: \MEXICO: Oaxaca, 52miles N Oaxaca, 9500 ft., 25 May 1971, ex. litter in sinkhole, S.Peck\ THOMAS C. BARR COLLECTION 2011 Acc. No. 38014\ (deposited in CAS, CMNH).

#### Specific epithet.

The specific epithet is a Latin adjective from *mons* (= mountain), in the masculine form, meaning *mountain-dwelling*, and refers to the altitudinal data of the species locality.

#### Type locality.

Mexico, Oaxaca, 52 miles N of Oaxaca.

**Recognition.** Males of this new species are distinguished from those of other species of the genus by the combination of elongate habitus and shape of the median lobe (Fig. 48).

#### Description.

*Size*. Medium-sized for genus (SBL range 1.29–1.40 mm, mean 1.35±0.037 mm, n=7).

*Habitus*. Body form (Fig. 29) slightly convex, moderately elongate (WE/SBL 0.40±0.10), head of normal proportions for genus (WH/WPm 0.75±0.041), pronotum narrow compared to elytra (WPm/WE 0.70±0.012).

*Color*. Body monocolorous, rufotestaceous, appendages testaceous.

*Microsculpture*. Partially effaced on disc of pronotum.

*Prothorax*. Pronotum relatively short (LP/LE 0.38±0.009) and moderately transverse (WPm/LP 1.26±0.028), with margins rectilinear and markedly constricted posteriorly (WPm/WPp 1.40±0.028). Basal margin oblique laterally. Contour of posterior angles obtuse (115–125°) with 1–2 small denticles in front of the angles.

*Elytra*. Convex, not depressed along suture, of moderate width (WE/LE 0.69±0.016). Margins subparallel at middle, slightly divergent in basal third, evenly rounded to apex in apical third, maximal width of elytra at midpoint.

*Legs*. 1^st^ male protarsomere markedly dilated apico-laterally.

*Male genitalia*. Median lobe of aedeagus (Fig. 48) with slightly elongate apex, angulately rounded at tip. Dorsal plate 1 short, pointed apically and basally. Dorsal plate 2 joined to plate 1 at its middle ventrally, where it forms a distinct protuberance. Ventral sclerites with pronounced sclerotization ventrally. Right paramere short and moderately wide (Fig. 50). Left paramere without apical constriction (Fig. 49). Ring sclerite with handle conically rounded apically (Fig. 65).

*Female genitalia*. Spermatheca standard for genus.

#### Geographical distribution.

The species is known only from the type locality in the Sierra Juárez Range, a part of the Sierra Madre de Oaxaca (Figs 77 and 94, white quadrangle).

#### Way of life.

Specimens of this species were collected at 2900–3000m, which is the highest locality known among the *Zapotecanillus* species. The collection site was located in a limestone area with sinkholes and karst topography, covered with a pine-oak forest. Soil temperature at the time of collection was 48°F (S.Peck, pers. comm.).

#### Relationships.

Externally, adults of *Zapotecanillus montanus* are similar to those of *Zapotecanillus kavanaughi*, *Zapotecanillus iviei* and *Zapotecanillus pecki*, but males of *Zapotecanillus montanus* may be distinguished from those of the other species by the shape of the median lobe (Fig. 48). See also Fig. 90 for cladistic affinities.

### Zapotecanillus pecki sp. n.

http://zoobank.org/AFF220F9-B257-44DF-8A08-F07F9E6988F3

http://species-id.net/wiki/Zapotecanillus_pecki

Figs 30 , 35 , 51–53 , 64 , 77 , 90 , 94

#### Type material.

HOLOTYPE, male, in CMNH, point-mounted, labeled: \ MEXICO, Oaxaca, 3.5miles S of Suchixtepec,\ Ber.208, leaf litter, 3 June 1971 S.B. Peck collector\ CMNH\. PARATYPES (16 ex., 4♂2♀ were dissected), 6 ex. labeled same as a holotype; 6 ex. labeled: \ MEXICO, Oaxaca, 13 mi. N of Suchixtepec, 9500ft., ex. leaf litter, 4 June 1971, S. Peck\ THOMAS C. BARR COLLECTION 2011 Acc. No. 38014\; 4 ex. labeled: MEXICO, Oaxaca, 13.5 mi. S of Suchixtepec, 8000ft., ex. leaf litter, 3 June 1971, S. Peck\ THOMAS C. BARR COLLECTION 2011 Acc. No. 38014\ (deposited in CAS, CMNH).

#### Specific epithet.

The specific epithet is a Latinized eponym in the genitive case, and is based on the surname of Stewart B. Peck, Professor in the Biology Department of Carleton University, Ottawa, Canada, the collector of the type series of this species.

#### Type locality.

Mexico, Oaxaca, 3.5miles S of Suchixtepec.

#### Recognition.

Males of this new species are distinguished from those of other species of the genus by the shape of the median lobe (Fig. 51).

#### Description.

*Size*. Medium-sized for genus (SBL range 1.32-1.38 mm, mean 1.35±0.026 mm, n=5).

*Habitus*. Body form (Fig. 30) slightly convex, moderately elongate (WE/SBL 0.41±0.08), head of normal proportions for genus (WH/WPm 0.73±0.011), pronotum narrow compared to elytra (WPm/WE 0.72±0.009).

*Color*. Body monocolorous, rufotestaceous, appendages testaceous.

*Microsculpture*. Microlines partially effaced on disc of pronotum.

*Prothorax*. Pronotum (Fig. 35) relatively short (LP/LE 0.41±0.012) and slightly transverse (WPm/LP 1.24±0.030), with margins slightly sinuate and distinctly constricted posteriorly (WPm/WPp 1.36±0.043). Basal margin bisinuate near posterior angles. Contour of posterior angles slightly obtuse (108–118°) with 1-2 small denticles in front of the angles.

*Elytra*. Slightly convex, not depressed along suture, of moderate width (WE/LE 0.70±0.022). Margins subparallel at middle, slightly divergent in basal half, evenly rounded to apex in apical half, maximal width of elytra at midpoint.

*Legs*. 1^st^ male protarsomere markedly dilated apico-laterally.

*Male genitalia*. Median lobe of aedeagus (Fig. 51), with elongate apex, rounded at tip. Dorsal plate 1 long, pointed apically and basally. Dorsal plate 2 joined to plate 1 at its apical third, where it forms a pronounced biapical protuberance. Ventral sclerite faintly sclerotized, barely visible. Right paramere rather long and moderately wide, with additional (3^rd^) seta dorsally (Fig. 53). Left paramere without apical constriction (Fig. 52). Ring sclerite with short handle, widely rounded apically (Fig. 64).

*Female genitalia*. Spermatheca standard for genus.

#### Geographical distribution.

The species is known only from the type locality in the Sierra Madre del Sur, in the surroundings of Suchixtepec (Figs 77 and 94, black flower).

#### Way of life.

Members of this species live at elevations of 8000-9500’ (2440-2900 m). At 8000’ (= 2440 m), beetles were collected in mixed pine-oak forest with *Alnus*, *Carpinus*, etc, and soil temperature at the time of collection was 56°F (S. B. Peck, pers. comm.).

#### Relationships.

Males of this species are easily distinguished from those of other members of the genus by the structure of the median lobe (Fig. 51) and setation of the right paramere (Fig. 53); and the geographical distribution of this species sets it apart from all its congeners. See also Fig. 90 for cladistic affinities.

### Zapotecanillus longinoi sp. n.

http://zoobank.org/778A7293-CE0E-4EBB-B2E2-AEE3CB9857B2

http://species-id.net/wiki/Zapotecanillus_longinoi

Figs 31 , 57–59 , 67 , 71 , 76 , 77 , 90 , 94

#### Type material.

HOLOTYPE, male, in KUNHM, point-mounted, labeled: \ MEXICO: Chiapas: Sierra Morena 16.16001°N, 93.60519°W, 1360m, 12-V-2008, ex. sifted leaf litter, 2° mesophil forest LLAMA08 Wa-A-01-1-all \ SM0833744 KUNHM-ENT \. PARATYPES (16 ex., 2♂3♀ were dissected), 9 ex. labeled same as a holotype, except barcode SM... numbers; 1 ex. labeled: \ MEXICO: Chiapas: Sierra Morena 16.15950°N, 93.60530°W, 1360m, 12-V-2008 sifted leaf litter, 2° mesophil forest LLAMA08 Wm-A-01-1 \ SM0839196 KUNHM-ENT \; 4 ex. labeled: \ MEXICO: Chiapas: Sierra Morena 16.15342°N, 93.60078°W, 1330m, 12-V-2008 ex. sifted leaf litter, 2° mesophil forest LLAMA08 Wa-A-01-2-all \ SM... KUNHM-ENT \; 2 ex. labeled: \ MEXICO: Chiapas, Mpio: Villa Corso, Ejido Sierra Morena, R. Biosfera La Sepultura, 16°09'10.6N, 93°35'25.1W, 1550m, 17–18.VII.2003, R. Anderson, mixed oak/pine forest litter, MEX1A03 110 \ SM... KUNHM-ENT \ (deposited in CAS, KUNHM).

#### Specific epithet.

The specific epithet is a Latinized eponym in the genitive case, and is based on the surname of John T. (Jack) Longino, Professor of the Biology Department of the University of Utah, and one of Co-PI’s of the LLAMA project, which provided the material on which the description of this species is based.

#### Type locality.

Mexico, Chiapas, Sierra Morena, 16.16001°N, 93.60519°W.

#### Recognition.

Adults of this new species can be distinguished from those of other species of the genus by the combination of small size and rufotestaceous color; and males can be further distinguished by the shape of the median lobe (Fig. 57).

#### Description.

*Size*. Small sized for genus (SBL range 1.01-1.12 mm, mean 1.08±0.038 mm, n=12).

*Habitus*. Body form (Fig. 31) slightly convex, slightly elongate (WE/SBL 0.40±0.10), head of normal proportions for genus (WH/WPm 0.76±0.021), pronotum moderately wide compared to elytra (WPm/WE 0.76±0.014).

*Color*. Body monocolorous, rufotestaceous, appendages testaceous.

*Microsculpture*. Partially effaced on disc of pronotum.

*Prothorax*. Pronotum relatively short (LP/LE 0.41±0.010) and moderately transverse (WPm/LP 1.28±0.035), with margins rectilinear and moderately constricted posteriorly (WPm/WPp 1.35±0.037). Basal margin oblique laterally. Contour of posterior angles obtuse (114–125°) with 0-1 small denticles in front of the angles.

*Elytra*. Slightly convex, not depressed along suture, of moderate width (WE/LE 0.69±0.015). Margins nearly subparallel, slightly divergent in basal forth, evenly rounded to apex in apical third, maximal width of elytra near midpoint.

*Legs*. 1^st^ male protarsomere markedly dilated apico-laterally.

*Male genitalia*. Median lobe of aedeagus (Fig. 57), with enlarged apex and neighboring part of ventral margin. Dorsal plate 1 small, pointed apically and narrowly rounded basally. Dorsal plate 2 located close to plate 1, in form of narrow stylet and shifted slightly apically. Ventral sclerites faintly sclerotized. Right paramere short and moderately wide (Fig. 59). Left paramere as in Fig. 58. Ring sclerite with short handle widely rounded apically (Fig. 67).

*Female genitalia*. Gonocoxite 2 comparatively short, with moderately curved blade and narrowly rounded apex (Fig. 71). Laterotergite with 7-8 setae. Spermatheca standard for genus (Fig. 76).

#### Geographical distribution.

The species is known only from the type locality in the Sierra Madre de Chiapas (Figs 77 and 94, black triangles).

#### Way of life.

According to the label data, these beetles inhabit mesophyll and mixed oak/pine forests at low elevations.

#### Relationships.

In the structure of median lobe of males and geographical distribution, this species is only remotely related to its congeners. See also Fig. 90 for cladistic affinities.

### *Zapotecanillus* sp.

Fig. 77

**Material.** MEXICO: Chiapas: 4km SE Custepec 15.71018°N, 92.92887°W, 2140m, 20-V-2008 ex. sifted leaf litter, cloud forest LLAMA08 Wa-A-03-1-all \ SM0832667 KUNHM-ENT \ (1♂); MEXICO: Chiapas: 4km SE Custepec 15.70673°N 92.93127°W, 2125m, 20-V-2008 ex: sifted leaf litter, cloud forest LLAMA08 Wa-A-03-2-all \ SM0821667 KUNHM-ENT \ (1♀), both in CAS.

Among the materials at hands, these two teneral specimens remain unidentified because of insufficient material for investigation. They were collected in the cloud forest near Custepec in the Sierra Madre de Chiapas (Fig. 77, white star). Both specimens resemble *Zapotecanillus longinoi* adults externally but are larger in size, and cannot be identified unambiguously. This locality represents the most southern point of the known range of *Zapotecanillus*.

Adults of the eight described species of this new genus are distinguished using the following key:

### Key for identification of the Mexican species of Zapotecanillus

**Table:** 

1	Small (1.00–1.20 mm in length). Sierra Madre de Chiapas and Sierra Madre de Oaxaca within Sierra de Juárez	2
–	Large (greater than 1.25 mm in length). Sierra Madre de Oaxaca and Sierra Madre del Sur	3
2	Darker, brunneorufous (Fig. 25). Apex of median lobe unmodified (Fig. 39). Dorsal plate 1 larger (Fig. 39). Sierra Madre de Oaxaca	*Zapotecanillus nanus*, p. 63
–	Lighter, rufotestaceous (Fig. 31). Apex of median lobe enlarged (Fig. 57). Dorsal plate 1 smaller (Fig. 57). Sierra Madre de Chiapas	*Zapotecanillus longinoi*, p. 77
3	Less robust and more elongate, body monocolorous, either brunneorufous (Fig. 26) or rufotestaceous (Figs 27–30). Pronotum narrower (WPm/LP < 1.30). Pronotal basal margin oblique (Figs 33–34) or sinuous laterally (Fig. 35)	4
–	More convex and robust, body with brunneorufous pronotum and rufotestaceous elytra (Fig. 24). Pronotum wider, distinctly transverse (WPm/LP 1.33±0.022). Pronotal basal margin straight (Fig. 32). Sierra de Juárez	*Zapotecanillus oaxacanus*, p. 61
4	Rufotestaceous beetles. Apex of median lobe elongate and narrow (Figs 45; 48, 51, 54). Dorsal plate 1 with long apical attenuation (Fig. 45), or pointed basally (Figs 48, 51, 54). Sierra Madre del Sur and Sierra Madre de Oaxaca within Sierra de Juárez and Sierra Aloapaneca	5
–	Brunneorufous beetles. Apex of median lobe short and broadly rounded (Fig. 42). Dorsal plate 1 long, rounded basally and with short apical attenuation (Fig. 42). Sierra de Juárez	*Zapotecanillus ixtlanus*, p. 65
5	Pronotal basal margin bisinuate laterally (Fig. 35). Dorsal plate 2 enlarged basally in form of biapical protuberance (Fig. 51). Sierra Madre del Sur	*Zapotecanillus pecki*, p. 74
–	Pronotal basal margin oblique laterally (Figs 33–34). Dorsal plate 2 of another shape. Sierra Madre de Oaxaca	6
6	Median lobe with elongate apex of normal size (Figs 45; 48). Spermatheca ball-shaped (Fig. 74). Sierra de Juárez	7
–	Median lobe with small and narrow apex (Fig. 54). Spermatheca fusiform with apical bulb enlargement (Fig. 75). Sierra Aloapaneca	*Zapotecanillus kavanaughi*, p. 69
7	Pronotal baso-lateral margin with weak sinuation before posterior angles (Fig. 34). Dorsal plate 1 greater, rounded basally and with long apical attenuation (Fig. 45). Cerro Pelon, Sierra de Juárez	*Zapotecanillus iviei*, p. 67
–	Pronotal baso-lateral margin rectilinear constricted towards posterior angles (as on Fig. 33). Dorsal plate 1 small, pointed basally and with rather short apical attenuation (Fig. 48). Sierra de Juárez to the South from Cerro Pelon	*Zapotecanillus montanus*, p. 71

### Results of cladistic analysis

**Figures 78–89.**
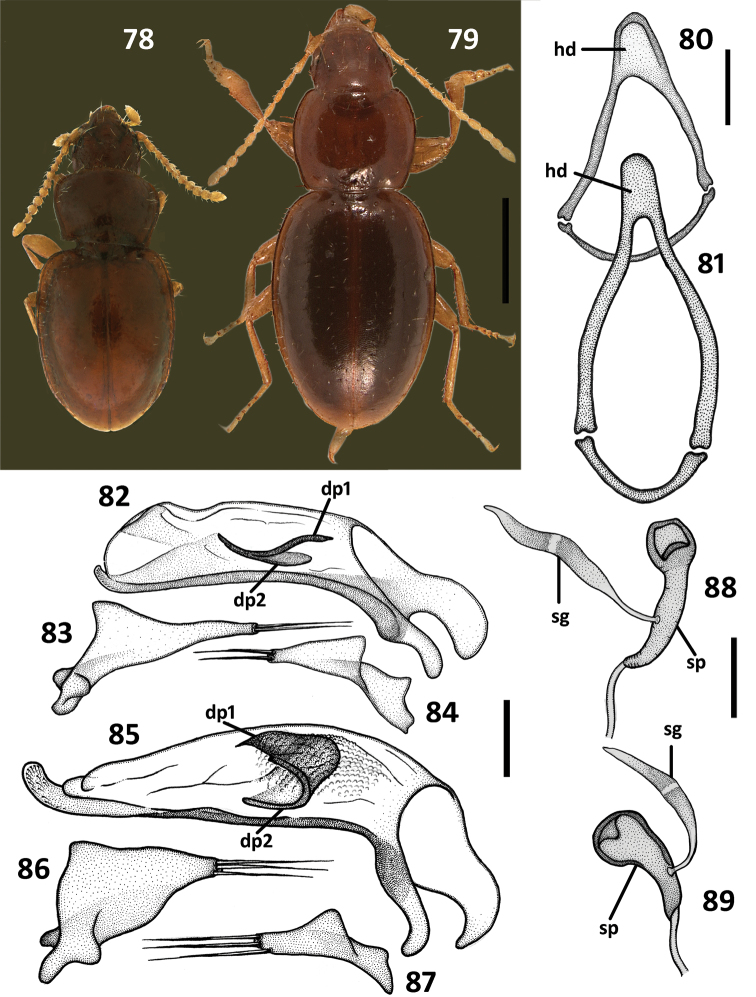
Structural features of the litter anilline species, chosen as an outgroup for the cladistic analysis of *Zapotecanillus* species. *Geocharidius phineus* Erwin **78** habitus **80** ring sclerite, dorsal aspect **82** median lobe, right lateral aspect **83** left paramere, left lateral aspect **84** right paramere, right lateral aspect **88** spermatheca. *Nesamblyops* sp. **79** habitus **81** ring sclerite, dorsal aspect **85** median lobe, right lateral aspect **86** left paramere, left lateral aspect **87** right paramere, right lateral aspect **89** spermatheca. dp1 – dorsal plate 1; dp2 – dorsal plate 2; hd – handle of round sclerite; sg – spermathecal gland; sp – spermatheca. Scale bars = 0.5mm (Figs **78–79**), 0.1mm (Figs **80–81**), 0.05mm (Figs **82–89**).

**Figures 90–94.**
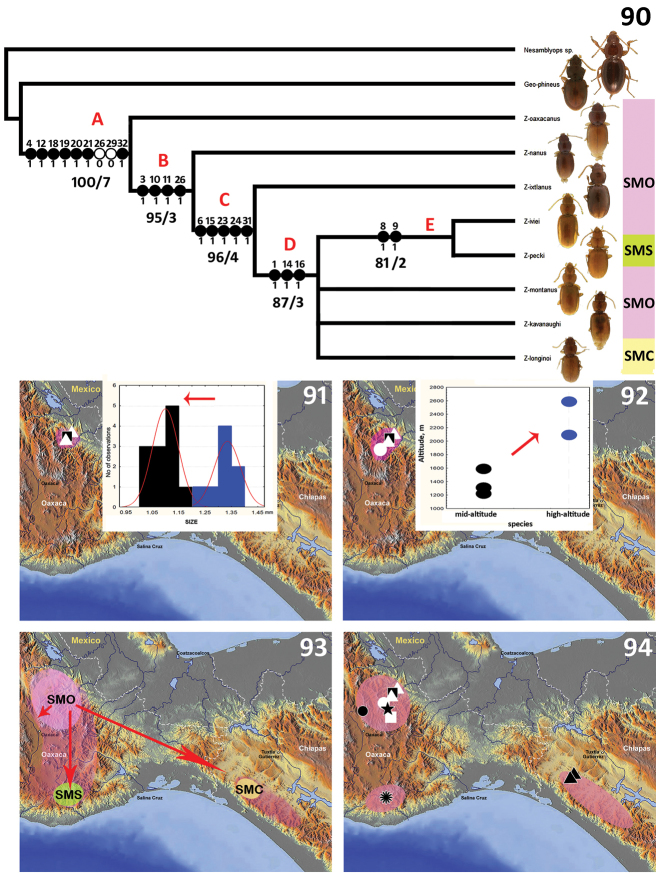
Cladistic relationships and main events of the natural history of *Zapotecanillus* species, inferred from the cladogram. **90** 75%-majority rule cladogram from the parsimony analysis: character states are shown only for nodes, forward changes as filled circles, and reversals as open circles; numbers under internal branches – indicate bootstrap/Bremer support indices; the letters B, C, and D above the nodes correspond to the maps **91, 92** and **93** below, illustrating evolutionary trends at the appropriate node; bar abbreviations, SMO – Sierra Madre de Oaxaca, SMS – Sierra Madre del Sur, SMC – Sierra Madre de Chiapas **91** syntopic miniaturization, blue bars – *Zapotecanillus oaxacanus*, black bars – *Zapotecanillus nanus* **92** altitudinal expansion, blue dots – *Zapotecanillus ixtlanus*, black dots – *Zapotecanillus oaxacanus* + *Zapotecanillus nanus* **93** regional dispersal, SMO – Sierra Madre de Oaxaca, SMS – Sierra Madre del Sur, SMC – Sierra Madre de Chiapas **94** modern distribution of species, *Zapotecanillus oaxacanus* – black quadrangle; *Zapotecanillus nanus* – white triangles; *Zapotecanillus ixtlanus* – white circle; *Zapotecanillus iviei* – black star; *Zapotecanillus kavanaughi* – black circle; *Zapotecanillus montanus* – white quadrangle; *Zapotecanillus pecki* – black flower; *Zapotecanillus longinoi* – black triangles.

The parsimony analysis resulted in two most parsimonious trees (L=53; CI=0.74; RI=0.76); the 75% majority-rule consensus cladogram of these trees is presented in Fig. 90, with the characters and support values mapped on the corresponding internal branches. The main basal nodes of the cladogram are highly supported by Bootstrap and Bremer indices, whereas a part of the terminal nodes is inadequately supported, which results in collapsed branches. The *Zapotecanillus* species form a well-supported monophyletic group (clade A, bootstrap value=100). Their monophyly is supported by the derived states for characters 4 (labial mental suture), 12 (additional apicolateral pronotal setae), 18 (positions of 7, 8, and 9 pores of umbilicate series), 19 (elytral subapical sinuation), 20 (shape of the intercoxal process of the abdomen), 21 (shape of the metendoventrite) and 32 (shape of the spermatheca). Within the genus, a basal clade is presented by *Zapotecanillus oaxacanus*, which is morphologically the closest species to the outgroup taxa (the latter selected from the litter species of anilline genera *Geocharidius* and *Nesamblyops*, Figs 78 and 79, respectively). Clade B is characterized by few traits, highlighting changes in the species’ appearance – notably the proportional reduction in the size of pronotum (character 3) and the shifting of the pronotal hind angles in a forward direction (characters 10 and 11); also the apex of median lobe is getting smaller (character 26). Clade C includes species with derived shared characters, which intensify the habitual dissimilarity with outgroup taxa. The pronotum in members of these species is proportionally shorter (character 6) and the elytra are narrower (character 15); also, internal parts of the male genitalia, namely the ring sclerite (characters 23 and 24), and parameres (character 31), are reduced. Clade D includes the species that are most unlike the basal taxa externally. Members of species in this clade have slightly convex bodies (character 1) and are completely yellow in color (characters 14 and 16). Members of Clade E species share a narrow pronotum with a shallow sinuation of the lateral margins anterior to the hind angles (characters 8 and 9). Thus, the cladogram of species’ relationships primarily reflects the gradual changes in external characteristics from basal to terminal clades, incorporating some changes in genitalic structures. The trend in changes in external form on the cladogram (from ovoid and pigmented towards elongate and depigmented beetles) reflects evolutionary adaptations for a more endogean way of life.

### Taxonomic and evolutionary issues

New data enable us to discuss several taxonomical and evolutionary issues, despite the limited material available for *Zapotecanillus*.

### Taxonomical notes

*Remarks on Geocharidius larva.* Ten years ago, a description of the first-instar larva of *Geocharidius* was published (Grebennikov 2002), and since then, it has remained the only larva of Anillina known from the New World. Material from the locality where the larva was collected also contained adults of Anillina and was labeled: “MEX: Oaxaca, 17.6mi S Ixtlán de Juárez...” (l.c.). The larva was identified as *Geocharidius* larva by association with adults, first by Vasily Grebennikov (Ottawa Plant Laboratory, Canadian Food Inspection Agency), and later by Terry Erwin (Smithsonian Institute), who approved the identification (l.c.). Based on data available now, *Geocharidius* species do not occur in the state of Oaxaca, whereas *Zapotecanillus* species do occur there. Thus, it is likely that the larva described in 2002 is of a *Zapotecanillus* species, rather than a *Geocharidius* species. Erwin did not distinguish the two genera, so his labeling the representatives of *Zapotecanillus* as *Geocharidius* sp. is understandable.

*Notes about Zapotecanillus.* As previously mentioned, it is difficult to assess relationships of the new genus to the other North and Central American Anillina without a modern revision of the latter. Therefore, the conclusions drawn below should be treated as preliminary and speculative.

Externally, the absence of discal setae is a feature that members of this new genus share with those of the southern stock of genera, like *Geocharidius*, *Mexanillus* and *Honduranillus* Zaballos (Zaballos 1997). The enigmatic *Honduranillus*, described from one female, is the only genus of Anillina in North and Central America whose members lack elytral discal pores but have distinct paraglossae, traits shared with *Zapotecanillus* members. However, the differences in arrangement of the last umbilicate pores and the length of the apical palpomere of the maxillae suggest that these similarities may be convergent. Historically, great importance has been given to the arrangement of setae in the apical portion of the umbilicate series. The above-mentioned southern stock of anilline genera belongs to the “scotodipnienne” evolutionary lineage, members of which have pores 7 and 8, and 8 and 9 separated from each other by equal distances (“Type B” of Jeannel’s classification), whereas *Zapotecanillus* is formally a representative of another evolutionary lineage of Anillina, the “anillienne” lineage, in which pores 8 and 9 are distinctly closer to each other than pore 7 is to pore 8 (the so-called“ geminate” arrangement, “Type A” of Jeannel (1963a)). Recently, Giachino and Vailati (Giachino and Vailati 2011), treating the anilline fauna of Greece, discovered that representatives of the “anillienne” genus *Prioniomus* Jeannel demonstrate great variation in the positions of pores 8 and 9. This discovery led the authors to propose a scheme of positional rearrangements of the setae of umbilicate series, leading from the “scotodipnienne” to the “anillienne” arrangement of pores. Similar rearrangements in the position of the 7^th^ through 9^th^ pores of the umbilicate series may well have occurred with the evolution of *Zapotecanillus* species. If so, *Zapotecanillus* may be the sister-taxon of *Geocharidius*; and the diversification of each genus may then be associated with one of two mountainous regions – namely, Oaxaca and Nuclear Middle America – which are separated by the Isthmus of Tehuantepec (Fig. 77), an important biogeographical barrier in the region under question (Ball 1968; Halffter 1987; Marshall and Liebherr 2000). In this case, the common ancestor of both genera would have been characterized as an anilline beetle with the “scotodipnienne” type of umbilicate series, as well as it would also lack discal pores on the elytra, and would have long last maxillary palpomeres, distinct paraglossae, free labial complex (i.e., mentum and submentum not fused), and simple metendoventrite. Subsequent evolution led to independent modifications of these traits and resulted in the origin of these two genera, members of which are morphologically very dissimilar. Presumably, we can tie the divergence of the two genera to the middle Pliocene, c. 3.1–3.5 Ma, when the Isthmus was replaced by a marine embayment (Barrier et al. 1998). Molecular analyses of many taxa among reptiles (Castoe et al. 2009; Daza et al. 2010), birds (Barber and Klicka 2010), and rodents (Hardy et al. 2013; Ornelas et al. 2013) provide evidence that this time is a historic milestone for Mesoamerican faunal diversification.

To confirm or reject the proposed phylogenetic relationship of *Zapotecanillus* within the North and Central American Anillina, the regional fauna requires further investigation, including analyses of additional morphological and, hopefully, molecular data.

### Evolutionary notes

Although cladistic analysis does not allow us to fully resolve phylogenetic relationships, some evolutional trends of *Zapotecanillus*, evident from the resulting cladogram, are worth examining.

Deviations from the form of the pronotum of litter species, such as the reduction in the overall size of the pronotum and forward shift of the hind angles (Fig. 90, clade B), reflect an increase in flexibility of the pronotal-elytral joint. A more flexible joint can potentially expand the number of accessible niches, enabling their bearers to live in a greater number of structurally different litter or soil interspace habitats. It seems that, among the litter-dwelling ancestors of *Zapotecanillus*, adaptations for living in a new environment were restricted by two major directions of species evolution.

The first direction, a syntopic habitat expansion, can be characterized as the intensification of local litter resource exploitation, presumably by means of niche differentiation. The structural complexity of the litter, undergoing decomposition, produces a graded series of overlapping planes interspersed with intertwined gaps, both of which tend to become smaller as one travels downwards toward the soil (Kaspari and Weiser 2007). In this case, new pronotal features enabling the species to move deeper, downwards through the intertwining environment, resulted in the differentiation of a miniature species *Zapotecanillus nanus* (Fig. 91), which co-occurred with its relatively larger and morphologically closest relative, *Zapotecanillus oaxacanus*. Syntopic miniaturization, producing a certain number of related species differing in size can be considered a common evolutionary trend within the anilline world. Pairs of syntopic small/large species pairs are known among the Central American *Geocharidius* (*Geocharidius phineus*–*Geocharidius romeoi* Erwin) (Erwin 1982), the North American *Anillodes* (*Anillodes walkeri* Jeannel-*Anillodes minutus* Jeannel) (Jeannel 1963a, 1963b), *Anillinus* (*Anillodes lescheni* Sokolov and Carlton – *Anillodes stephani* Sokolov and Carlton) (Sokolov et al. 2004), and the European *Typhlocharis* Dieck (Pérez-Gonzáles and Zaballos 2013a). In some cases, even more than two species of anillines may be syntopic, as it was shown for the Pyrenean *Typhlocharis* Dieck, among which three species, well-differentiated in size, co-occur (Pérez-Gonzáles and Zaballos 2013b). The evolution of *Zapotecanillus longinoi*, another miniature species of *Zapotecanillus*, also matches the proposed scheme of evolution, except that, in this case, the role of the large species is played by a representative of another genus, *Geocharidius*.

The second direction of evolution among *Zapotecanillus* species was connected with the altitudinal expansion of the genus and subsequent adaptations to the endogean way of life. In Clade C (Fig. 90), pronotal and elytral morphology have undergone additional modifications. Most of the species of this clade live at elevations above 2400m, as exemplified by *Zapotecanillus ixtlanus* (Fig. 92). The ability to live at high elevation implies adaptations to withstand daily and seasonal variations in temperature and humidity. One solution to this challenge is the acquisition of adaptations that facilitate vertical migrations, from the litter down into the soil and back to the litter again to track favorable and escape from unacceptable microclimatic shifts (e.g., regular frosts at high elevations of the Sierra de Juárez). Also, changes in forest communities along the elevation gradient can play an additional role in the evolution of species adaptations. In the Sierra Madre de Oaxaca, the humid montane cloud forests at elevations of 1200–1600m are characterized by the dominance of broadleaf tree species, such as *Quercus*, *Liquidambar*, *Carpinus*, and *Fagus* (Flores and Manzanero 1999), while in the temperate high-elevation forests at altitudes of 2200m to 2800m, the dominant tree species are various species of *Pinus* (Peterson et al. 2004, Saynes et al. 2012), presumably with corresponding changes in the forest litter composition and structure. Such features of high-altitude anillines as elongate habitus together with small pronotum with an oblique basal margin, may represent adaptations to differences in the important climatic and vegetation parameters along the altitudinal gradient such as those mentioned above. Changes in the states of characters in Clade D (Fig. 90), including depigmentation and flattening of the body, may reflect and support the transition from the litter to the endogean way of life among the high-altitude *Zapotecanillus*. The same situation was recorded for the high-altitude Appalachian *Anillinus moseleyae*-group of species, Sokolov 2011). All terminals of clade D on the cladogram (Fig. 90) are represented by species (Figs 27–31) whose morphology suggests an endogean way of life (at least temporarily); and in one case (*Zapotecanillus montanus*) we have a straight reference to habitat (“sink litter”), which can be treated as support for the proposed speculations.

### Distributional notes

A review of the overall distribution of genus *Zapotecanillus* shows that species with endogean lifestyles are distributed across the whole range of the genus (Fig. 90, SMS, SMO, SMC), while litter-dwelling species are restricted to only the eastern slopes of the Sierra Madre de Oaxaca (Fig. 90, SMO). *A priori*, one might expect that litter-dwelling forms would be more broadly distributed than endogean forms, but within *Zapotecanillus*, this does not appear to be so. Perhaps additional litter-dwelling species of the genus remain undiscovered or have gone extinct in this region, but the extensive overall distribution of endogean species clearly suggests a role for them in the expansion and the shape of the modern geographical range of the genus. For instance, in the Sierra Madre de Chiapas, adults of *Zapotecanillus longinoi* and *Zapotecanillus* sp., both of the endogean morphological type, were collected at altitudes of 1330m to 2140m, which are approximately the same elevations at which low-altitude *Zapotecanillus oaxacanus* and *Zapotecanillus nanus* were collected in the Sierra de Juárez (Sierra Madre de Oaxaca). If a litter-dwelling ancestral *Zapotecanillus* species had dispersed from the Sierra Madre de Oaxaca to the Sierra Madre de Chiapas, one would expect members of the Chiapan descendant species to be similar in life-style and appearance to those of the Oaxacan ancestral form because low elevations are primarily occupied by litter species. However, adults of *Zapotecanillus longinoi* and *Zapotecanillus* sp., are flattened and depigmented, and quite different from the convex and pigmented litter-dwelling *Zapotecanillus* forms, as well as from litter-dwelling *Geocharidius* species. It is perhaps significant that *Zapotecanillus longinoi* and *Zapotecanillus* sp. members are syntopic with litter-dwelling species of *Geocharidius* in the Sierra Madre de Chiapas. It may be that the presence of two species of *Zapotecanillus* with endogean morphology at low elevations in the Sierra Madre de Chiapas represents a secondary occupation of the region by endogean forms, thus, supporting the idea that at least some endogean *Zapotecanillus* forms are capable of significant dispersal.

If we consider that the endogean way of life of the *Zapotecanillus* species was triggered by changes in microclimate parameters, then regional dispersal of the depigmented and only slightly convex *Zapotecanillus* species also could have been connected with certain climate changes. Such dispersal likely occurred during one or more of the Pleistocene glacial cycles, which enabled species with endogean life-styles to cross the Isthmus of Tehuantepec, and, perhaps, the Central Valleys of Oaxaca, and to establish populations in the Sierra Madre de Chiapas and the Sierra Madre del Sur, respectively (Fig. 93). Evidence that the Isthmus served as a corridor connecting Oaxaca and Chiapas Sierras during Pleistocene glaciations has been shown for some bird species (García-Moreno et al. 2004; Barber and Klicka 2010) as well as cloud forest communities (Ornelas et al. 2013). Regional dispersal of the ancestral *Zapotecanillus* stock eventually resulted in allopatric, presumably Quaternary, speciation in the genus, thereby shaping the modern distribution of the genus (Fig. 94).

The described sequence of events and exact mountain regions where the evolution and differentiation of *Zapotecanillus* took place are still debatable, given the paucity of data about the distribution and diversity of the genus and, thus, should be the subject of further investigations. For instance, the fauna of anillines is still unknown for such biogeographically important mountain ranges in Oaxaca as the Sierra de Zempoaltepec and the Sierra de Mijes. It is also still unknown how far to the north and west across the Oaxacan highlands *Zapotecanillus* species are distributed. These and many other questions await further investigation and discoveries.

## Supplementary Material

XML Treatment for Zapotecanillus

XML Treatment for Zapotecanillus oaxacanus

XML Treatment for Zapotecanillus nanus

XML Treatment for Zapotecanillus ixtlanus

XML Treatment for Zapotecanillus iviei

XML Treatment for Zapotecanillus kavanaughi

XML Treatment for Zapotecanillus montanus

XML Treatment for Zapotecanillus pecki

XML Treatment for Zapotecanillus longinoi

## Appendix

**Table 1.** Morphological characters and states used for cladistic analysis.

*External characters*	
1.	Body shape. 0, moderately convex (Figs 24–26, 78–79); 1, slightly convex (Figs 27–31).
2.	Standardized body length. 0, larger, > 1.20mm; 1, smaller, <1.20mm.
3.	Relative head width, ratio WH/WPm. 0, head narrower, with ratio <0.70; 1, head wider, with ratio > 0.71.
4.	Labial mental suture. 0, present (Fig. 8); 1, absent (Fig. 7).
5.	Paraglossae of ligula. 0, present (Fig. 7); 1, absent (Fig. 8).
6.	Relative pronotal length, ratio LP/LE. 0, pronotum longer, ratio > 0.42; 1, pronotum shorter, ratio < 0.42.
7.	Relative pronotal width, ratio WPm/WE. 0, pronotum wider, ratio > 0.80; 1, pronotum narrower, ratio < 0.78.
8.	Pronotal width, ratio WPm/LP. 0, more transverse, ratio > 1.25; 1, less transverse, ratio < 1.25.
9.	Pronotal basolateral sinuation. 0, absent (Figs 32–33); 1, shallow (Figs 34–35).
10.	Pronotal basal margin. 0, straight (Fig. 32); 1, laterally oblique (Figs 33–34); 2, laterally sinuous (Fig. 35).
11.	Pronotal hind angles. 0, nearly rectangular, <110⁰ (Figs 32, 35); 1, slightly obtuse, >115⁰ (Figs 33–34).
12.	Additional apicolateral pronotal setae. 0, absent (Fig. 2); 1, present (Fig. 1).
13.	Pronotal discal microsculpture. 0, with distinct mesh microsculpture; 1, with obsolete microsculpture, at most represented by fine parallel lines without mesh formation.
14.	Pronotal coloration. 0, brown; 1, yellowish.
15.	Elytral width, ratio WE/LE. 0, wider, ratio >0.71; 1, narrower, ratio <0.71.
16.	Elytral coloration. 0, brown; 1, yellowish.
17.	Discal pores on elytra. 0, present; 1, absent.
18.	Position of 7, 8, and 9 pores of umbilicate series. 0, equidistant (Fig. 4); 1, 8 and 9 pores close to each other than to 7 (Fig. 3).
19.	Elytral preapical sinuation. 0, elytra evenly tapered (Fig. 4); 1, elytra slightly sinuate subapically (Fig. 3).
20.	Intercoxal process of 2^nd^ ventrite. 0, triangular (Fig. 14); 1, apically truncated (Fig. 13).
21.	Shape of metendosternite. 0, simple, without “arms” (Fig. 14); 1, cross-shaped, with long lateral “arms” (Fig. 13).
22.	Male protarsomere. 0, noticeably expanded, with modified setae (Figs 20–21); 1, barely expanded, with modified setae (Fig. 22); 2, not expanded, without modified setae (Fig. 23).
*Male genitalic characters*	
23.	Length of a handle of ring sclerite. 0, long (Figs 60–61, 66, 80–81), 1, short (Figs 62–65, 67).
24.	Shape of a handle of ring sclerite. 0, subparallel (Figs 60–61, 66, 80–81); 1, conical (Figs 62–65, 67).
25.	Median lobe shape. 0, bent dorsad from basal opening (Figs 36, 39, 42, 45, 48, 51, 85); 1, bent at basal opening (Figs 54, 57, 82).
26.	Contour of the apex of median lobe, a lateral view. 0, straight, short, of moderate width (Figs 36, 42, 57); 1, straight, slightly elongated, of moderate width (Figs 39, 45, 48, 51); 2, straight, elongated and very narrow (Fig. 54); 3, upcurved, elongated and narrow (Figs 82, 85).
27.	Modification of neighboring parts of apex of median lobe. 0, absent (Figs 36, 39, 42, 45, 48, 51, 54, 82, 85); 1, enlarged ventrally (Fig. 57).
28.	State of dorsal plate 1, a lateral view. 0, long plate with rounded basal part (Figs 36, 39, 42, 45); 1, short plate with rounded basal part (Fig. 57); 2, long plate with pointed basal part (Fig. 51); 3, short plate with pointed basal part (Figs 48, 54); 4, long and curved stylet-shaped plate (Fig. 82); 5, triangular plate (Fig. 85).
29.	State of dorsal plate 2, a lateral view. 0, adjoined to dorsal plate 1, like a small knob-like protuberance (Figs 36, 39, 42, 45, 48); 1, adjoined to dorsal plate 1, like a large biapical protuberance (Fig. 51); 2, like a separate structure either crossing or parallel to dorsal plate 1 (Figs 54, 57); 3, attached to dorsal sclerite 1 in form of a long appendix (Figs 82, 85).
30.	Right paramere, seta number. 0, 3 setae (Figs 53, 87); 1, 2 setae (Figs 38, 41, 44, 47, 50, 56, 59, 84).
31.	Left paramere, shape of apical third. 0, with attenuated apex (Figs 37, 40, 83, 86); 1, with evenly tapering sides to the tip (Figs 43, 46, 49, 52, 55, 58).
*Female genitalic characters*	
32.	Spermatheca, shape. 0, elongated with apical bulb enlargement (Figs 75, 88–89); 1, spherical, ball-like (Figs 72–74, 76).

**Table 2.** Data matrix used for cladistic analysis.

										***1***	***1***	***1***	***1***	***1***	***1***	***1***	***1***	***1***	***1***	***2***	***2***	***2***	***2***	***2***	***2***	***2***	***2***	***2***	***2***	***3***	***3***	***3***
	***1***	***2***	***3***	***4***	***5***	***6***	***7***	***8***	***9***	***0***	***1***	***2***	***3***	***4***	***5***	***6***	***7***	***8***	***9***	***0***	***1***	***2***	***3***	***4***	***5***	***6***	***7***	***8***	***9***	***0***	***1***	***2***
*Nesamblyops* sp.	0	0	0	0	0	0	0	0	0	0	0	0	1	0	0	0	0	0	0	0	0	0	0	0	0	3	0	5	3	0	0	0
*Geocharidius phineus*	0	0	0	0	1	0	1	0	0	0	0	0	1	0	0	0	1	0	0	0	0	2	0	0	1	3	0	4	3	1	0	0
*Zapotecanillus oaxacanus*	0	0	0	1	0	0	0	0	0	0	0	1	0	0	0	1	1	1	1	1	1	1	0	0	0	0	0	0	0	1	0	1
*Zapotecanillus nanus*	0	1	1	1	0	0	1	0	0	1	1	1	1	0	0	0	1	1	1	1	1	0	0	0	0	1	0	0	0	1	0	1
*Zapotecanillus ixtlanus*	0	0	1	1	0	1	1	0	0	1	1	1	0	0	1	0	1	1	1	1	1	0	1	1	0	0	0	0	0	1	1	1
*Zapotecanillus iviei*	1	0	1	1	0	1	1	1	1	1	1	1	1	1	1	1	1	1	1	1	1	0	1	1	0	1	0	0	0	1	1	1
*Zapotecanillus montanus*	1	0	1	1	0	1	1	0	0	1	1	1	1	1	1	1	1	1	1	1	1	0	1	1	0	1	0	3	0	1	1	1
*Zapotecanillus kavanaughi*	1	0	1	1	0	1	1	1	0	1	1	1	1	1	1	1	1	1	1	1	1	2	0	0	1	2	0	3	2	1	1	0
*Zapotecanillus pecki*	1	0	1	1	0	1	1	1	1	2	0	1	1	1	1	1	1	1	1	1	1	0	1	1	0	1	0	2	1	0	1	1
*Zapotecanillus longinoi*	1	1	1	1	0	1	1	0	0	1	1	1	1	1	1	1	1	1	1	1	1	0	1	1	1	0	1	1	2	1	1	1
